# Enhanced Azo Dye Removal via Alginate-Immobilized *Ganoderma lucidum*: From Batch Experiments to Continuous Column Studies

**DOI:** 10.3390/polym18192317

**Published:** 2026-09-22

**Authors:** Aslı Göçenoğlu Sarıkaya

**Affiliations:** Department of Chemistry, Bursa Uludağ University, 16059 Bursa, Turkey; agocenoglu@uludag.edu.tr; Tel.: +90-224-295-50-73

**Keywords:** *Ganoderma lucidum*, Direct Blue 9, biosorption, alginate microbeads, fungal biomass

## Abstract

In this study, *Ganoderma lucidum* biomass and alginate-immobilized *G. lucidum* microbeads were compared for the removal of the azo dye Direct Blue 9 (DB9) from aqueous solutions. While raw fungal biomass has been widely studied as a biosorbent, its practical application is limited by poor mechanical stability and reusability. To overcome these limitations, biomass was immobilized via alginate microencapsulation, and its performance was systematically compared with free biomass. The effects of pH, temperature, initial DB9 concentration, and contact time were optimized for both systems. The immobilized microbeads exhibited a significantly higher removal efficiency (99.54 ± 0.76%) than free biomass (93.31 ± 0.05%), indicating a positive effect of immobilization on biosorption performance. Equilibrium data were best described by the Freundlich isotherm model, suggesting heterogeneous surface interactions, while thermodynamic analysis confirmed that the process was exothermic and occurred spontaneously. Reusability tests were performed, and the microbeads maintained high removal efficiency across multiple cycles with ethanol as the desorption agent, highlighting their operational stability. Furthermore, continuous column studies confirmed the applicability of immobilized biosorbents under dynamic conditions, demonstrating their potential for scale-up. Results indicate that alginate-immobilized *G. lucidum* microbeads provide an efficient and practical alternative to free biomass for dye removal in wastewater treatment systems.

## 1. Introduction

The presence of persistent organic pollutants in various environmental matrices is a major concern worldwide. The limited availability and poor quality of water resources, driven by the release of diverse anthropogenic pollutants, constitute a serious threat to human health and aquatic ecosystems [1,2]. Therefore, the reuse of treated wastewater has emerged as a practical and promising solution to meet current global water requirements. Among these pollutants, synthetic dyes and dyestuffs originating from industrial wastewater streams heavily contaminate groundwater and clean water sources. Azo dyes, in particular, account for approximately 60–70% of the dyes utilized in industry [3]. Due to their resonance structures and extensive π-conjugations, most azo dyes exhibit high resistance to degradation by heat, light, water, detergents, or bleaching agents.

Direct Blue 9 (DB9) is one of the azo dyes commonly used in the textile industry, containing two azo bonds (-N=N-) attached to aromatic rings [4]. These dyes possess mutagenic and carcinogenic effects in humans and some animals, transforming into electrophilic compounds that form covalent bonds with DNA and disrupt the DNA double helix by cleaving the aromatic amine at the azo bond [5]. DB9 is highly soluble in water and organic solvents such as ethanol.

Conventional chemical or physical treatment of dye removal wastewater is restricted by the complex molecular structures of these compounds. Consequently, bioremediation has attracted considerable interest as an eco-friendly and cost-effective alternative in wastewater treatment. This approach utilizes microorganisms, including bacteria, yeasts, filamentous fungi, and microalgae, to catalyze the degradation or transformation of toxic chemicals into less harmful forms [6]. Bioremediation strategies are generally classified into active/passive biosorption and enzymatic approaches [7]. In passive biosorption, the use of inactive/dead cells as biosorbents enables the binding of specific ions or molecules in wastewater via ion exchange, adsorption, surface complexation, and other mechanisms. This mechanism represents an inexpensive, recyclable, reusable, and passive accumulation process [8].

Microbial biomass can be used in wastewater treatment either in free form or immobilized onto polymeric support materials [9]. Immobilization offers distinct operational advantages, such as easy separation, improved operational stability, multiple use, incorporation into fixed- and fluid-bed columns, and higher efficiency [10]. To ensure cost-effective production, various natural or synthetic polymers bearing reactive functional groups can serve as non-toxic carriers through techniques such as entrapment, cross-linking, covalent bonding, adsorption, and encapsulation [11,12]. Retaining microbial cells within such polymeric matrices not only enhances their biosorption capacity (*q_e_*) but also facilitates practical operational control.

*Ganoderma lucidum*, a medicinal mushroom, is rich in phenolic compounds, sterols, β-glucans, and terpenoids, traditionally cultivated for its antioxidant, immunomodulatory, anticancer, and anti-inflammatory properties [13,14]. Furthermore, it is widely used for the extracellular production of laccase, a ligninolytic enzyme [15,16]. However, the fungal biomass remaining after extracellular enzyme production is typically disposed of as a waste by-product. Reusing this spent biomass as a biosorbent offers a sustainable and circular economy approach. While numerous studies have demonstrated the efficacy of fresh *G. lucidum* biomass for pollutant removal [17,18,19], very few have explored the reuse of spent pellets derived from separate fermentation lines. In a study by Salvi et al., *Rhizopus arrhizus* pellets were used for biotransformation studies and subsequently for the treatment of azo dyes [20]. In another study, the fungus *Phanerochaete chrysosporium* was used to remove copper metal after its use in the synthesis of selenium nanoparticles and in the treatment of selenium-containing wastewater [21]. Similarly, *P. chrysosporium* was used as a biosorbent to remove Methylene Blue after its use in the treatment of refinery wastewater [22].

In this study, an integrated and sustainable process was developed by repurposing *G. lucidum* pellets, which was obtained as a spent waste by-product from a separate laccase enzyme production line, as a low-cost biosorbent for dye removal. Rather than evaluating this waste biomass solely in static batch configurations, the fungal pellets were successfully entrapped within a non-toxic and biodegradable calcium-alginate matrix. Crucially, the objective of this immobilization strategy extends far beyond merely confirming the obvious enhancement of mechanical strength or structural stability. Instead, this matrix was utilized as a practical engineering tool to transition the technology into continuous-flow, fixed-bed column systems. By systematically analyzing the dynamic breakthrough curves under different flow rates, bed heights, and dye concentrations, the real-world operating parameters needed for industrial-scale treatment systems were provided, fundamentally differentiating this approach from conventional, stand-alone biosorption studies.

## 2. Materials and Methods

### 2.1. Materials

Potato dextrose agar and malt extract broth were purchased from Condalab (Madrid, Spain). Direct Blue 9 was obtained from Sigma-Aldrich (St. Louis, MO, USA), and all other chemicals were of analytical grade.

### 2.2. Preparation of G. lucidum Biomass and Alginate-Immobilized G. lucidum Microbeads

*G. lucidum* was collected from Bursa (Türkiye), isolated, identified at Ege University, Department of Biology (İzmir, Türkiye), and stored at Bursa Uludağ University Department of Chemistry (Biochemistry/Biotechnology Research Laboratory) [23]. The stock cultures (Stock Number: Gl-B24) were cultivated on potato dextrose agar for 5 days at 28 °C. The mycelia were transferred to malt extract broth, which contained glucose (10 g/L) as a carbon source, yeast extract (5 g/L) as a nitrogen source, peptone (5 g/L), MgSO_4_.7H_2_O (5 g/L), CuSO_4_.5H_2_O (0.05 g/L), and incubated at 120 rpm at 28 °C for 10 days. After incubation, the pellets were filtered from the culture medium containing the laccase enzyme and washed three times with distilled water. The pellets were inactivated by autoclaving at 121 °C for 15 min and dried at 40 °C in an oven. The dried pellets were crushed into smaller pieces and stored as biosorbents at 4 °C for further studies.

For the synthesis of alginate-immobilized *G. lucidum* microbeads (*G. lucidum*@Alg), a sodium alginate solution at a concentration of 1–5% (*w*/*v*) was dissolved in 100 mL of distilled water. Subsequently, 0.5 g of *G. lucidum* biomass was added, and the mixture was magnetically stirred for 2 h to obtain a homogeneous suspension. This mixture was then added dropwise into a 0.1 M CaCl_2_ solution and stirred gently at room temperature until equilibrium was reached to ensure complete cross-linking. To remove excess cross-linker, the synthesized microbeads were filtered and thoroughly washed with distilled water. The resulting microbeads were transferred to clean Petri dishes, dried at room temperature, and stored in glass bottles for further biosorption studies.

### 2.3. Characterization of Alginate-Immobilized G. lucidum Microbeads

In order to determine the functional groups and surface morphology of the alginate-immobilized *G. lucidum* microbeads, Fourier transform infrared (FTIR) and Scanning Electron Microscopy (SEM) analyses were performed before and after biosorption using a PerkinElmer Spectrum 100 FT-IR spectrometer (PerkinElmer, Shelton, CT, USA) and GeminiSEM 3000 instrument (ZEISS, Oberkochen, Germany), respectively.

To calculate the gel fraction (*F_gel_*) of the microbeads prepared in different formulations, the samples were initially dried at 40 °C for 4 h to determine their initial dry weight (*W*_0_) and subsequently immersed in deionized water for 24 h. The water-swollen microbeads were then dried again under the same conditions (40 °C for 4 h) to obtain the final dry weight (*W_e_*). The *F_gel_* value was calculated according to Equation (1) [24]:

(1) $$F_{gel}=\frac{W_{e}}{W_{0}}\times 100$$

To determine the water content, prepared microbeads were weighed in their wet state (*W_wet_*) after gently removing excess surface water, and then dried at 40 °C until a constant weight (*W_dry_*) was achieved. The water content of the microbeads (*C_w_*) was calculated according to Equation (2) [25]:

(2) $$C_{w}=\frac{W_{wet}-W_{dry}}{W_{dry}}$$

The swelling percentage (*Swelling*%) of microbeads prepared in different formulations was calculated according to Equation (3) [26]:

(3) $$Swelling\%=\frac{W_{wet}-W_{dry}}{W_{dry}}\times 100$$

To determine the physical dimensions of the synthesized biosorbents, 50 randomly selected wet microbeads from the optimum 3% formulation were measured using a digital caliper to find the average bead size. Additionally, to ensure the reproducibility of the synthesis protocol and account for batch-to-batch variation, three entirely independent batches of microbeads were prepared under identical conditions. These separate batches were then evaluated under standard biosorption conditions to confirm that the material properties remained consistent across different preparation times.

The point of zero charge (*pH_pzc_*) of the alginate-immobilized *G. lucidum* microbeads was determined using the salt addition method. The microbeads were introduced into NaCl (0.01 N) solutions with initial pH values ranging from 2.0 to 9.0, followed by stirring at 120 rpm at room temperature for 24 h. Thereafter, the final pH values of the solutions were recorded, and the *pH_pzc_* was determined by plotting Δ*pH* against the initial pH according to Equation (4) [27,28].

(4) $$\Delta pH={pH}_{final}-{pH}_{initial}$$

Liquid-phase Methylene Blue (MB) biosorption experiments were performed to determine the pore accessibility, specific surface area, and surface heterogeneity of the synthesized 3% alginate-immobilized *G. lucidum* microbeads in an aqueous solution [29]. The experiments were carried out in an aqueous medium based on the principle of isotherm modeling to eliminate the risk of pore shrinkage in biopolymeric gels caused by conventional gas adsorption techniques (BET) during drying. The biosorption experiments were performed in a batch system using an MB solution with an initial concentration of 20–200 mg/L at pH 6.0. 0.5 g of microbeads were added to 50 mL volumes of the dye solution, and the mixture was agitated at 120 rpm at 25 °C for 24 h. At the end of the incubation period, the MB concentrations at equilibrium were measured spectrophotometrically at 664 nm. All experiments were performed in triplicate (n: 3). The equilibrium biosorption capacities (*q_e_*, mg/g) of the microbeads were calculated according to Equation (5).

(5) $$q_{e}=\frac{\left(C_{0}-C_{e}\right)\times V}{m}$$

*C*_0_ and *C_e_* in the equation represent the MB concentration (mg/L) at the initial and equilibrium states, respectively; *V* represents the total volume of the MB solution (L); and *m* represents the amount of biosorbent used (g).

The maximum monolayer adsorption capacity (*q_max_*) obtained from the Langmuir isotherm model was used to calculate the specific surface area of the microbeads in the aqueous phase according to Equation (6).

(6) $$S_{MB}=\frac{q_{max}\times a_{MB}\times N_{A}}{M_{W}}\times 10^{-20}$$

*a_MB_* is the surface area covered by a MB molecule, *N_A_* is the Avogadro number (6.022 × 10^23^ mol^−1^), and *M_W_* is the molecular weight of MB (319.86 g/mol).

### 2.4. Removal of DB9 from Aqueous Solutions with G. lucidum Biomass and Alginate-Immobilized G. lucidum Microbeads

After determining the optimum formulation, a comprehensive biosorption study was conducted to evaluate the efficacy of the alginate-immobilized *G. lucidum* microbeads for dye removal from aqueous solutions, using DB9 as the target contaminant. To investigate the effect of pH on the process, biosorption experiments were performed within the pH range of 2.0–9.0 to identify the optimum operating pH. These batch experiments were carried out at 25 °C for 2 h in an orbital shaker incubator at 150 rpm, utilizing an initial DB9 concentration of 100 mg/L. Following the determination of the optimum pH, the influence of the initial dye concentration was systematically evaluated. To this end, biosorption tests were performed across an initial DB9 concentration range of 50–200 mg/L at three distinct temperatures (25, 35, and 45 °C). Furthermore, comparative biosorption studies were conducted using free *G. lucidum* biomass to benchmark the performance of the developed microbeads. The equilibrium biosorption capacity (*q_e_*, Equation (7)) and the biosorption percentage % (*B*%) (Equation (8)) were calculated using the following equations:

(7) $$q_{e}=\frac{\left(C_{0}-C_{e}\right)}{m}\times V$$

(8) $$Biosorption\;\%=\frac{C_{0}-C_{e}}{C_{0}}\times 100$$

*C*_0_ and *C_e_* in the equation represent the dye concentration (mg/L) at the initial and equilibrium states, respectively; *V* represents the total volume of the dye solution (L); and *m* represents the amount of biosorbent used (g). Color measurements were performed using a UV-visible spectrophotometer (*λ_max_*: 568 nm). All experiments were carried out in duplicate, and the data were reported as mean values with their corresponding standard deviations (±SD). Control runs were simultaneously conducted using dye solutions without any biosorbent to monitor potential dye degradation, and no significant changes in concentration were observed. Furthermore, blank experiments were performed using raw alginate microbeads under identical operational conditions. The dye removal efficiency of these blank microbeads was below 5%, confirming that the biosorption process was driven primarily by the active surface sites of the immobilized *G. lucidum* biomass rather than by the polymer matrix alone. The initial solution pH was adjusted with dilute HCl or NaOH, and final pH checks were performed after equilibrium to ensure no significant pH drift occurred during the process.

### 2.5. Desorption and Reusability

To select the most effective desorption agent, ethanol and methanol were evaluated for the desorption of DB9. The DB9 free *G. lucidum* biomass and alginate-immobilized *G. lucidum* microbeads were individually transferred into the respective desorption eluents and agitated in an orbital shaker incubator at 150 rpm for 5 h at 25 °C. Thereafter, the desorption ratio was calculated using Equation (9):

(9) $$Desorption\;ratio\%=\frac{Q_{d}}{Q_{b}}\times 100$$

*Q_b_* and *Q_d_* are the biosorbed and desorbed amounts of DB9, respectively. To assess the reusability of the biosorbents, biosorption–desorption cycles were performed five times using the same biosorbents and a suitable desorption agent.

### 2.6. Continuous Column Studies

The effective application of biosorbents in real-world scenarios is highly important. The performance of continuous column studies provides insight into the feasibility of using biosorbents in large-scale processes [4]. Hence, to evaluate this applicability, continuous column studies were conducted using the alginate-immobilized *G. lucidum* microbeads. The experiments were carried out in borosilicate glass columns with an inner diameter of 1.5 cm and a total height of 20 cm, packed uniformly with the alginate-immobilized *G. lucidum* microbeads. The key operational parameters, including packing height (3–7 cm), flow rate (3–7 mL/min), and initial DB9 concentration (50–200 mg/L), were optimized under various conditions. The column system was operated in a down-flow mode at pH 6.0 and 25 °C using a peristaltic pump. The DB9 solution was introduced into the column from the top, and effluent samples collected from the bottom were measured spectrophotometrically to monitor the breakthrough behavior.

### 2.7. Statistical Analysis

All biosorption and desorption experiments were performed in triplicate, and data were presented as mean ± standard deviation (SD). To confirm the reproducibility and reliability of the data, statistical analysis was carried out using the open-source statistical software Jamovi (Version 2.4). The significance of the performance gap between free and alginate-immobilized *G. lucidum* microbeads was evaluated using One-Way and Two-Way Analysis of Variance (ANOVA), followed by Tukey’s post-hoc test. A *p*-value of less than 0.05 (*p* < 0.05) was considered statistically significant, while *p* < 0.001 indicated high statistical significance.

## 3. Results

### 3.1. Characterization of G. lucidum Biomass and Alginate-Immobilized G. lucidum Microbeads

FTIR analyses were performed before and after biosorption to clarify the chemical structure and functional groups of *G. lucidum* biomass and alginate-immobilized *G. lucidum* microbeads to elucidate the interaction mechanisms between *G. lucidum* biomass, alginate-immobilized microbeads, and Direct Blue 9 dye (Figure 1). Accordingly, characteristic absorption bands of hydroxyl groups were observed around 3300 cm^−1^, with a noticeable change in intensity after biosorption, suggesting their involvement in hydrogen-bonding interactions [30]. Similarly, variations in the peaks at 2900 cm^−1^ and 1640 cm^−1^ were assigned to aliphatic C-H stretching, and Amide I (C=O) vibrations of the fungal biomass, respectively, indicate their participation in dye binding [31,32]. These spectral changes collectively suggest that multiple functional groups contribute to biosorption through combined hydrogen bonding and electrostatic interactions, although further surface charge analysis would strengthen interpretation [33,34]. After the biosorption of DB9, notable modifications occurred in the spectra of both the *G. lucidum* and alginate-immobilized microbeads. The reduction in peak intensity and broadening in the hydroxyl/amino region signify the active role of hydrogen bonding during dye uptake. Furthermore, the shifts observed in the Amide/carboxylate peak strongly indicate electrostatic interactions between the cationic active sites of the sorbents and anionic sulfonate groups of the DB9 dye molecule.

SEM images were used to determine the surface morphologies of *G. lucidum* biomass and alginate-immobilized microbeads (Figure 2). While free biomass exhibited a relatively compact structure, alginate-immobilized microbeads displayed a highly porous and heterogeneous surface. This structural modification is expected to enhance dye accessibility by improving mass transfer within the biosorbent matrix [35].

The water content and swelling percentage of 3% alginate microbeads with 0.5 g *G. lucidum* were 18.37 and 93.61%, respectively (Table 1). The swelling ratio of microbeads is essential for water molecule penetration and for facilitating dye diffusion into their internal structure. Swelling-induced porosity plays a critical role in enhancing biosorption performance, consistent with findings in hydrogel-based biosorption systems [36]. Overall, immobilization not only modifies surface morphology but also improves internal diffusion pathways, thereby enhancing dye uptake efficiency.

The 3% (*w*/*v*) alginate concentration was chosen for all subsequent experiments because it offered the ideal balance between physical durability and efficient dye diffusion, as demonstrated by the data in Table 1. This specific formulation achieved the highest gel fraction (*F_gel_* = 99.86%), maximum water content (*C_w_* = 18.37%), and peak swelling percentage (93.61%). Having a high swelling capacity is essential because it opens up the internal pore network of the microbeads, making it much easier for DB9 molecules to diffuse inside. When we tested higher alginate levels (4% and 5%; (*w*/*v*)), the polymer network became overly dense and rigid, which restricted swelling and blocked the diffusion pathways. Conversely, lower concentrations (1% and 2%; (*w*/*v*)) were structurally too weak to stably hold the high load of *G. lucidum* biomass. Consequently, the 3% (*w*/*v*) formulation stood out as the optimal choice for ensuring both structural integrity and maximum performance. For further biosorption studies, 3% (*w*/*v*) alginate was used as a biosorbent.

The 3% (*w*/*v*) alginate concentration was selected for all subsequent experiments because it provided the most suitable balance between structural durability and efficient dye diffusion, as demonstrated by the systematic data presented in Table 1. This specific formulation achieved the highest gel fraction (*F_gel_* = 99.86%), optimum water content (*C_w_* = 18.37%), and peak swelling percentage (93.93%). A high swelling capacity is considered essential in practice, as the internal polymeric network expands to facilitate the intraparticle diffusion of DB9 molecules into the deeper active sites of the matrix. When higher alginate levels (4% and 5%, *w*/*v*) were evaluated, the polymer network became overly dense and rigid, which restricted the swelling ratio and blocked the internal diffusion pathways, leading to a decline in performance. Conversely, lower concentrations (1% and 2%, *w*/*v*) were found to be structurally too weak to securely entrap the 0.5 g load of *G. lucidum* biomass, resulting in lower gel fractions and potential matrix instability.

The *pH_pzc_* value was determined to understand the possible interactions of the functional groups on alginate-immobilized *G. lucidum* microbeads with metal species in solution, as well as their ionization. The *pH_pzc_* value of the microbeads was calculated as 5.20 (Figure 3), and as the pH increased, the surface charge of the microbeads became negatively charged. *G. lucidum* biomass is rich in chitin, chitosan, protein, and glucans. The mannuronic acid and guluronic acid units in the alginate structure are rich in hydroxyl and carboxyl groups. The *pK_a_* value of alginate is approximately 3.5 [37]. When the pH is below 4, all carboxyl groups in the alginate are protonated; the microbeads shrink, their pores close, and they become hydrophobic. Similarly, the *pH_pzc_* value of alginate-clay bio-nanocomposites was found as 6.2 [38]. It is believed that minor changes in *pH_pzc_* may occur when other biomasses are added to the alginate matrix.

To characterize the surface area and pore accessibility of the synthesized 3% alginate-immobilized *G. lucidum* microbeads, the MB sorption method in the aqueous phase was used. The equilibrium data at pH 6.0 were analyzed according to the Langmuir isotherm model. Based on the capacity value calculated from the Langmuir isotherm model (*q_max_* = 464.19 mg/g), the specific surface area (*S_MB_*) of the microbeads was determined as 1136.16 m^2^/g. The high surface area value obtained indicates that the synthesized biosorbent possesses a high level of pore and empty space volume. Furthermore, the high retention capacity of the cationic dye (MB) serves as experimental evidence that the microbeads carry a very high concentration of active negative charge at pH 6.0.

### 3.2. Biosorption Studies

The pH of the medium is an important parameter for biosorption processes because it affects the ionic form of the biosorbent and the dye present in the medium. Variations in pH affect the binding sites of the dye and biosorbents [39,40]. Protonatable functional groups such as amino, carboxyl, hydroxyl, and phosphate groups are present in fungal biomass [41,42]. As the pH value of the medium changes, the charge of these functional groups also changes. At a medium pH around 6, amino groups become protonated, and the biosorbent surface becomes positively charged. Azo dyes are mostly anionic at pH 6, leading to strong electrostatic interactions with the biosorbent and thereby enhancing biosorption. At low pH, H^+^ ions and anionic dye molecules in solution compete for active sites on the biosorbent surface, thereby decreasing biosorption, as H^+^ ions can bind to these sites [34]. At high pH, deprotonation of carboxyl groups renders the biosorbent surface negatively charged, hindering dye binding [43,44,45]. A similar situation applies to fungal biomass embedded in alginate. At approximately pH 5, the carboxylate groups in the alginate structure are protonated, resulting in a balanced surface charge. According to biosorption results, the *q_e_* for *G. lucidum* biomass and alginate-immobilized microbeads at pH 6 were 15.548 ± 0.034 and 34.278 ± 1.102 mg/g, respectively. Biosorption percentages were determined 77.317 ± 0.034 and 85.228% ± 1.102 for *G. lucidum* biomass and alginate-immobilized microbeads, respectively (Figure 4). At around pH 6, the carboxyl groups are completely deprotonated; the negative charges repel each other, and the microbeads begin to swell. Consequently, DB9 molecules begin to enter the microbeads. The amino groups in the chitin and chitosan structures found in the cell wall of *G. lucidum* are partially protonated at pH 6. As the microbeads expand at pH 6, DB9 easily diffuses into the matrix; it is believed that electrostatic interactions and strong hydrogen bonds form between the fungal biomass and DB9. Based on this, the biosorption mechanism may involve electrostatic interactions, hydrogen bonding, ion exchange, and surface complexation [35].

A Two-Way ANOVA was used to statistically compare free (*G. lucidum*) and alginate-immobilized *G. lucidum*. According to the results (Table S1), the biomass type heavily influences the qe values (*F*_(1,32)_ = 32,686, *p* < 0.001). Both the solution pH (*F*_(7,32)_ = 4605, *p* < 0.001) and the interaction between biomass type and pH (*F*_(7,32)_ = 554, *p* < 0.001) were also highly significant. These statistical results clearly prove that alginate-immobilized *G. lucidum* microbeads perform mathematically better and remain more stable than free biomass at every pH level tested.

The effect of initial DB9 concentration and temperature on biosorption was evaluated at three different temperatures (25, 35, and 45 °C) within the range of 50–200 mg/L. The increase in biosorption efficiency as temperature decreases is related to biosorption kinetics [46]. Furthermore, at low concentrations, the active sites on the biosorbent surface become saturated with dye molecules; as the concentration increases, the diffusion rate increases, and biosorption occurs more rapidly. Once a certain concentration is reached, no significant increase in biosorption capacity is observed due to saturation of the biosorbent’s active sites [47]. According to the results, both *q_e_* and *B*% increased with increasing concentration and decreased with increasing temperature (Figure S1). The highest *q_e_* were found at 25 °C for an initial DB9 concentration of 200 mg/L, with 36.798 ± 0.795 mg/g for *G. lucidum* biomass and 89.50 ± 0.01 mg/g for alginate-immobilized microbeads, respectively. The fact that *q_e_* and *B*% are higher at lower temperatures suggests that the biosorption process is exothermic (Figure 5).

As given in Figure 4, a decrease in both *q_e_* and *B*% values was observed with increasing temperature from 25 to 45 °C. Chemically, this temperature-dependent trend is directly related to the exothermic nature of the biosorption process, which is firmly verified by the negative standard enthalpy change (Δ*H*°) [48]. However, this downward trend cannot be attributed solely to thermodynamic equilibrium forces; it is also strongly influenced by physicochemical changes within the calcium-alginate carrier matrix under thermal stress [49]. With increasing temperature, the solubility of the adsorbate increases, thereby enhancing the adsorbate-adsorbent interaction forces due to the Brownian movement of the adsorbate particles in the liquid medium. Consequently, as temperature rises, the chemical potential decreases and the biosorption capacity declines [50]. The high positive entropy change (Δ*S*°) values obtained in this system indicate that rising temperatures induce physical adjustments in the immobilized microbeads, such as polymer chain relaxation, de-swelling, or the release of clustered water molecules from the hydrogel cavities [51]. These structural modifications alter the orientation and accessibility of the embedded *G. lucidum* active sites, thereby contributing to reduced biosorption performance at higher temperatures. Consequently, the effect of temperature on the system is governed by a dual mechanism involving both the exothermic energy profile of the bonding process and the simultaneous structural alterations of the carrier microbeads.

Furthermore, a clear distinction must be made between the decrease in experimental equilibrium capacities (*q_e_*) at a fixed concentration shown in Figure 4 and the significant increase observed in the theoretical maximum monolayer capacities (*Q_L_*) derived from the Langmuir model in Table 2. While the exothermic nature of the process lowers real-time dye uptake at a given concentration, the temperature-induced matrix relaxation simultaneously unlocks and exposes a greater total number of potential active sites at saturation. Therefore, the seemingly contrasting trends between the experimental performance profiles and the theoretical maximum model limits are not contradictory and reflect a well-balanced interplay between exothermic binding thermodynamics and physical expansion of the hydrogel matrix [52].

In addition, biosorption experiments using alginate were also performed to identify the individual and synergistic effects of alginate and fungal biomass (Figure S2).

To evaluate how temperature and immobilization simultaneously affect the final biosorption performance, a Two-Way ANOVA was applied to the equilibrium data at the 180th minute. The statistical output confirmed that the immobilization process remains the dominant factor driving the *q_e_* values (*F*_(1,12)_ = 33,140.5, *p* < 0.001). While temperature also significantly influenced the system (*F*_(2,12)_ = 589.2, *p* < 0.001) and showed a meaningful interaction with the biomass type (*F*_(2,12)_ = 74.3, *p* < 0.001), the post-hoc Tukey test revealed that the alginate-immobilized *G. lucidum* microbeads maintained a remarkably higher and more stable capacity than free biomass across all tested temperatures (25, 35, and 45 °C). The results were also given at Table S2.

### 3.3. Desorption and Reusability

Regeneration and reusability represent a critical parameter for evaluating the practical applicability of biosorbents in continuous wastewater treatment systems. Long-term operational stability is considered essential for ensuring cost-effectiveness and minimizing secondary waste generation in industrial-scale applications.

In this study, ethanol and methanol were evaluated as desorption agents. In the initial screening runs, a desorption efficiency of 90% was achieved with ethanol, whereas methanol achieved only 42.73%. Consequently, ethanol was selected as the sole eluent for all subsequent regeneration cycles. This markedly higher desorption performance of ethanol can be observed due to its lower dielectric constant and higher hydrophobic character compared to methanol, which altogether weakens the non-covalent interactions, such as hydrogen bonding and hydrophobic forces, formed between the *G. lucidum* functional structures and the DB9 fractions [53]. Beyond chemical extraction, ensuring that the organic solvent does not damage the polymer structure during recycling is vital [54]. Calcium-alginate hydrogels do not dissolve in ethanol; on the contrary, ethanol acts as an anti-solvent that triggers phase separation, causing the alginate polymer chains to shrink and dehydrate slightly (de-swelling) [51]. This structural tightening plays a protective role, preventing the microbeads from eroding, degrading, or leaking the entrapped biomass [55]. Consequently, the alginate-*G. lucidum* matrix fully maintains its structural integrity and mechanical durability over multiple reuse cycles without any visible deformation. Every desorption cycle was monitored by weighing the dried microbeads, and no significant mass loss (<1%) or biomass leaching was observed due to the excellent gel fraction (99.86%) of the 3% alginate matrix.

Ethanol was used as a desorption agent to regenerate both *G. lucidum* biomass and alginate-immobilized microbeads over five successive biosorption–desorption cycles. Ethanol effectively disrupts weak physical interactions, such as hydrogen bonding and electrostatic attractions, between DB9 molecules and biosorbent functional groups, thereby facilitating dye release without causing severe structural damage.

The results showed that the initial desorption efficiencies were 82.137 ± 0.949% for biomass and 95.209 ± 0.894% for immobilized beads, confirming highly efficient regeneration in the first cycle (Figure 6). After five cycles, the efficiencies decreased to 55.269 ± 0.954% and 71.326 ± 0.841%, respectively. The higher retention of sorption capacity and structural durability observed in the alginate-immobilized microbeads suggests that the cross-linked polymeric matrix provides mechanical shielding for the embedded fungal structures, thereby limiting the loss of active binding environments during repeated exposures to fluid stress and organic solvents. Consequently, the developed composite microbeads demonstrate acceptable operational durability for cyclical dye remediation processes. Optical images of alginate-immobilized *G. lucidum* before and after biosorption, and after desorption processes, are shown in Figure S3.

A Two-Way ANOVA was conducted to evaluate the reusability profiles of free and alginate-immobilized *G. lucidum* over five consecutive desorption cycles. The statistical results revealed that the immobilization process had a highly significant positive effect on maintaining high desorption efficiencies (*F*_(1,20)_ = 12,910.3, *p* < 0.001). While the desorption efficiency naturally declined across subsequent cycles for both forms (*F*_(4,20)_ = 5542.0, *p* < 0.001), the interaction between biomass type and cycle number was also significant (*F*_(4,20)_ = 25.7, *p* < 0.001). These findings mathematically verify structural stability and regeneration capacity than the free biomass throughout multiple cycles. The results were also given at Table S3.

### 3.4. Isotherm, Kinetic, and Thermodynamic Parameters

Biosorption isotherms were calculated to clarify the interaction behavior between the biosorbent and dye ions [56]. To investigate the biosorption process, Langmuir, Freundlich, and Dubinin–Radushkevich (D-R) isotherm models were utilized, and the corresponding parameters are compiled in Table 2. The Langmuir isotherm model typically applies to uniform, single-layer systems with a limited biosorption capacity [57]. *C_e_* (mg/L) represents the concentration of DB9 in the solution at equilibrium after biosorption, *q_e_* (mg/g) represents the amount of DB9 biosorbed, *Q_L_* (mg/g) represents the maximum biosorption capacity, and *K_L_* represents the Langmuir constant. *K_L_* and *Q_L_* were calculated using Equation (10).

(10) $$\frac{C_{e}}{q_{e}}=\frac{1}{Q_{L}K_{L}}+\frac{C_{e}}{Q_{L}}$$

The Freundlich isotherm model describes processes on heterogeneous surfaces with varying biosorption energies [58] and is given by Equation (11). *K_F_* (mg/g) represents the biosorption capacity, *q_e_* (mg/g) represents the amount of DB9 biosorbed onto the biosorbent at equilibrium, *C_e_* (mg/L) is the dye concentration in the solution at equilibrium, and *n* is the Freundlich isotherm constant. When *n* < 1, biosorption is a physical property [59].

(11) $$\ln q_{e}=\ln K_{F}+\frac{1}{n}\ln C_{e}$$

The Dubinin–Radushkevich (D-R) isotherm model, developed by incorporating the porous structure of the biosorbent, accounts for potential changes on heterogeneous surfaces [60] and is calculated using Equation (12).

(12) $$\ln Q_{e}=\ln Q_{D-R}-K_{D-R}\varepsilon^{2}$$

*Q_e_* gives the amount of biosorbed substance, *K_D-R_* gives the D-R constant, and *Q_D-R_* gives the maximum biosorption capacity (mg/g). *ε* Polanyi potential (J/mol) is expressed and calculated as given in Equation (13).

(13) $$\varepsilon =RT\ln\left(1+\frac{1}{C_{e}}\right)$$

*R* (J/mol K) is the universal gas constant. *C_e_* is the equilibrium concentration of the biosorbate (mg/L), and *T* is the temperature (K). The free energy of biosorption (*E_fe_*) is calculated from the *K_D-R_* value (Equation (14)).

(14) $$E_{fe}=\frac{1}{\sqrt{-2K_{D-R}}}$$

The equilibrium biosorption data were analyzed using Langmuir, Freundlich, and Dubinin–Radushkevich (D-R) isotherm models (Table 2) to elucidate the nature of the interaction between the DB9 dye molecules and the biosorbent surfaces. The Langmuir isotherm model represents monolayer adsorption on a homogeneous surface, whereas the Freundlich model assumes multilayer adsorption on heterogeneous surfaces with non-uniform energy distribution [56,57]. In this study, the Freundlich model provided a highly satisfactory fit for both *G. lucidum* biomass and alginate-immobilized *G. lucidum* microbeads, as reflected by the higher correlation coefficients (*R*^2^) across the investigated temperatures. This agreement suggest that the biosorption process occurs predominantly on energetically heterogeneous sites rather than forming a strictly uniform monolayer. This surface heterogeneity is attributed to the presence of diverse functional groups originating from both the fungal cell wall network and cross-linked alginate matrix, which altogether create multiple distinct binding environments for DB9 molecules. The calculated Freundlich intensity parameters (*n* < 1) mathematically indicate a cooperative or joint multi-site binding nature on these heterogeneous composite surfaces [46]. Furthermore, the calculated Langmuir separation factor (*R_L_*) values fell between 0 and 1, confirming that the biosorption process remains thermodynamically favorable over the temperature range studies [61].

The D-R isotherm model, developed by incorporating the porous structure of the biosorbent, was further employed to gain preliminary insights into the apparent adsorption energy (*E_fe_*). The calculated mean free energy values were found to be below 8 kJ/mol (*E_fe_* < 8 kJ/mol), which suggests that physical interactions, including electrostatic attraction, ion exchange, and hydrogen bonding, play a significant role during the initial uptake stages [62,63]. However, given the structural and chemical complexity of the composite framework, the overall mechanism cannot be definitively classified as purely physical based on a single model fit. Instead, a combined mechanism involving physical diffusion and joint multi-site surface interactions is highly anticipated.

Kinetic studies represent a key parameter for understanding the biosorption mechanism and identifying the potential rate-limiting steps [64]. The biosorption kinetics of DB9 onto both *G. lucidum* biomass and alginate-immobilized *G. lucidum* microbeads were evaluated using pseudo-first-order, pseudo-second-order, and intraparticle diffusion models (Table 3).

A pseudo-first-order kinetic model can be determined using Lagergren’s first-order rate equation [63] (Equation (15)).

(15) $$\ln\left(q_{e}-q_{t}\right)=\ln q_{e}-k_{1}t$$

When the pseudo-first-order kinetic model is insufficient, the pseudo-second-order kinetic model is used [65] (Equation (16)). The *q_t_* and *q_e_* values (mg/g) represent the amount of dye biosorbed by the biosorbent at any time *t* and at equilibrium, respectively, while *k*_1_ (1/min) represents the equilibrium rate constant. *k*_2_ (g/mg min) is the pseudo-second-order rate constant.

(16) $$\frac{t}{q_{t}}=\frac{1}{k_{2}q_{e}^{2}}+\frac{t}{q_{e}}$$

The intra-particle diffusion model was calculated using Equation (17), which includes the velocity constant, *k_id_*.

(17) qt=kidt12

The adsorption kinetics of DB9 onto both *G. lucidum* biomass and alginate-immobilized microbeads were evaluated using pseudo-first-order, pseudo-second-order, and intraparticle diffusion models (Table 3).

As represented in Table 3, the experimental kinetic profiles showed a highly satisfactory mathematical agreement with pseudo-second-order model, yielding superior correlation coefficients (*R*^2^) compared to the pseudo-first-order model under different thermal conditions. While a high fit to the pseudo-second-order kinetic model frequently points toward chemical surface interactions involving electron sharing or electrostatic coupling with surface functional groups (such as carboxyl, hydroxyl, and amine groups), a definitive chemical bond formation or pure chemisorption mechanism cannot be inferred from kinetic parameters alone without independent [66,67]. Therefore, kinetic behavior is interpreted more cautiously as a combined resistance, where both surface functional group interactions and mass transfer resistance govern the overall adsorption rate.

To further investigate the specific diffusion pathways, the intraparticle diffusion model was applied. The resulting linear plots did not pass through the origin, which clearly confirms that intraparticle diffusion is not the sole rate-controlling step. Consequently, boundary layer diffusion and external surface binding are considered to contribute simultaneously to the overall rate-limiting framework of the biosorption process, signifying that surface interactions play a primary role rather than simple pore diffusion alone.

The consistency of pseudo-second-order kinetics with fungal-based biosorbents reported in the literature supports the reliability of the obtained results. A comparison of different biosorbents (Table 4) further confirms that both free and immobilized *G. lucidum* systems exhibit competitive performance in azo dye removal.

Kinetic analysis suggests that the biosorption process is primarily controlled by surface interactions rather than simple diffusion, with both surface binding and pore diffusion contributing to the overall rate.

Thermodynamic analyses determine whether biosorption occurs spontaneously and describe the physical or chemical properties of the process. Δ*G*° indicates that a process occurs spontaneously. If Δ*G*° is negative, it indicates that biosorption occurs spontaneously and is energetically favorable [72]. The increase in adsorption performance at lower temperatures further supports the exothermic nature of the process. Δ*H*° indicates that the biosorption process is endothermic (positive Δ*H*°) or exothermic (negative Δ*H*°) [73,74]. Additionally, if Δ*H*° is less than 40 kJ/mol, it indicates physical biosorption [75,76]. This suggests that the interaction between Direct Blue 9 molecules and biosorbent functional groups is mainly governed by weak forces, such as electrostatic attraction and hydrogen bonding, rather than by strong chemical bonding, consistent with the FTIR and kinetic results. A positive Δ*S°* value indicates an increase in randomness at the interface between the biosorption surface and the dye solution [77]. This behavior can be attributed to the displacement of water molecules from the biosorbent surface and increased disorder at the interface upon dye binding. The higher Δ*S°* observed for immobilized microbeads suggests that the alginate matrix facilitates greater structural flexibility and enhanced interaction sites.

The enthalpy (Δ*H*°), free energy (Δ*G*°), and entropy (Δ*S°*) change values were calculated using the van’t Hoff equation (Equation (18)). The Δ*G*° is calculated using Equation (19).

(18) $$\ln K_{L}=-\frac{\Delta H{}^{\circ}}{RT}+\frac{\Delta S{}^{\circ}}{R}$$

(19) ΔG°=ΔH°−TΔS

*T* is the absolute temperature in Kelvin, *R* is the universal gas constant, and *K_L_* is the Langmuir constant.

Thermodynamic parameters for the biosorption of DB9 onto *G. lucidum* biomass and alginate-immobilized microbeads were evaluated at 25, 35, and 45 °C (Table 5) to investigate the energetic feasibility and spontaneity of the process. The negative standard Gibbs free energy (Δ*G*° < 0) values observed across all investigated temperatures indicate that the biosorption process is spontaneous and thermodynamically favorable for both biosorbents [72].

The enthalpy changes (Δ*H*° = −56.450 kJ/mol for biomass and −68.635 kJ/mol for immobilized microbeads) confirm that biosorption is exothermic. The relatively high magnitude of Δ*H*° suggests that the process involves strong interactions between the dye molecules and the active functional groups on the biosorbent surface, rather than solely weak physical adsorption. This is consistent with combined electrostatic interactions and hydrogen bonding mechanisms observed in FTIR analysis.

As presented in Table 5, the standard Gibbs free energy change (Δ*G*°) values range from −105.273 to −108.550 kJ/mol for raw *G. lucidum* and exhibit even larger negative values from −134.167 to −138.565 kJ/mol for the alginate-immobilized microbeads. While large negative changes in free energy are frequently associated with heavily driven surface interactions in complex composite matrices, a definitive assignment of an absolute pure chemisorption mechanism cannot be validated based on these apparent thermodynamic parameters alone. The energetic profile is governed by a combined contribution from both negative enthalpy (Δ*H*° < 0) and positive entropy (Δ*S°* > 0) factors. The positive standard entropy changes (Δ*S°* = 163.836 J/mol K for biomass and 219.905 J/mol K for microbeads) reflect an increase in randomness at the solid–liquid interface during the biosorption process, which is commonly associated at the solid–liquid interface during the biosorption process, which is commonly associated with the release of hydrated water molecules from dye ions upon binding to the heterogeneous functional groups. Therefore, rather than indicating a single isolated mechanism, the thermodynamic parameters suggest a highly cooperative and multi-site binding environment enhanced by the alginate immobilization framework.

### 3.5. Continuous Column Studies

Continuous column experiments were conducted to investigate the feasibility of using alginate-immobilized microbeads for large-scale biosorbent applications under dynamic flow conditions. The influence of bed height, flow rate, and initial DB9 concentration on column performance was systematically investigated.

Bed height significantly affected column performance by altering the available binding sites and length of the mass transfer zone. Therefore, dye removal experiments were performed at bed heights ranging from 3 cm to 7 cm. The initial DB9 concentration was adjusted to 50 mg/L. Increasing bed height increased contact time between the dye solution and active functional groups, leading to improved bed utilization and enhanced biosorption capacity. This behavior suggests a more efficient distribution of the adsorption front within the column, indicating improved fixed-bed adsorption performance at higher bed depths (Figure 7).

The flow rate of the dye through the column is another important parameter affecting biosorption capacity. Flow rate experiments demonstrated that lower flow rates enhanced biosorption efficiency by increasing residence time and reducing external film diffusion resistance. Therefore, DB9 was passed through the column at different flow rates (3, 5, 7 mL/min). The results showed that decreasing the flow rate increased biosorption capacity. At higher flow rates, insufficient contact time between the dye molecules and the biosorbent surface led to earlier breakthrough, indicating mass transfer limitations under fast flow conditions. The results are shown in Figure 8.

Breakthrough curve analysis at different initial concentrations (50–200 mg/L) revealed that higher inlet concentrations led to earlier breakthrough and column saturation due to rapid occupation of active sites. DB9 was passed through a column with a bed height of 7 cm at a flow rate of 3 mL/min. In contrast, lower concentrations allowed for more efficient bed utilization and extended breakthrough time, reflecting improved adsorption efficiency under diluted conditions (Figure 9).

Overall, the column studies demonstrate that the biosorption performance is strongly influenced by hydrodynamic conditions and mass-transfer limitations. The observed breakthrough behavior suggests that the system could offer a useful baseline for continuous wastewater treatment investigations. However, rather than acting as a definitive framework for industrial upscaling, these results highlight a fundamental starting point that requires further optimization through mathematical breakthrough modeling and pilot-scale testing under realistic operational setups.

### 3.6. Industrial Applicability and Spent Sorbent Management

From an industrial perspective, destroying dyes in wastewater through chemical reactions often requires excessive energy and risks creating even more toxic by-products. In contrast, the alginate-immobilized *G. lucidum* system developed in this work offers a much safer, eco-friendly, and cost-effective alternative by simply transferring the pollutant from water to a solid matrix. The economic side of the process is highly practical: instead of growing fungal biomass from scratch, the *G. lucidum* pellets were obtained as a free by-product from a separate laccase enzyme production line. This integrated approach ensures a virtually cost-free raw material strategy, making the entire system highly sustainable.

The evaluation of this technology was conducted through continuous fixed-bed column operations rather than limiting the scope to simple laboratory beakers. By analyzing the dynamic breakthrough profiles across various bed heights, flow rates, and dye concentrations, useful preliminary indicators of the dynamic behavior of the microbeads were identified, which may assist in defining baseline parameters for future scale-down pilot studies.

To follow the principles of a circular economy and prevent secondary pollution, the dye-loaded microbeads are not meant to be thrown away after a single use. Instead, the trapped DB9 dye can be efficiently stripped and concentrated using ethanol, allowing the beads to be reused over multiple treatment cycles. Crucially, the recovered dye molecules in the concentrated desorption eluent can be directly repurposed as a secondary raw material for re-dyeing fresh textile fabrics, significantly reducing the demand for pristine commercial dyestuffs [78]. This circular approach aligns with sustainable waste-to-resource strategies documented in the literature, in which regenerated effluent systems are reintegrated into textile coloration processes without compromising fabric quality. At the end of their lifespan, the exhausted beads can be safely managed either by burning them in waste-to-energy plants to generate electricity or by incorporating them into solid construction materials such as cement or bricks. Through these practical disposal routes, any final threat to soil or water ecosystems is completely eliminated.

Furthermore, the economic and practical feasibility of the immobilization process must be evaluated by examining how the system performs in real-world scenarios. Even though free *G. lucidum* pellets show a high dye removal efficiency on their own (~92%), using them directly in large-scale treatment plants is practically impossible. In a continuous-flow system, loose fungal pellets quickly pack together, clogging the treatment towers and blocking water flow. Therefore, trapping the biomass inside alginate microbeads is not just to achieve a minor efficiency boost; it is a necessary step to keep the water flowing smoothly without clogging.

From an economic standpoint, preparing these composite microbeads does require a small initial expense for sodium alginate and calcium chloride. However, this cost is quickly recovered due to two major advantages. First, the core raw material (*G. lucidum* pellets) is a free waste by-product left over from laccase enzyme production. Second, unlike free biomass, which is usually discarded after a single use, the protective alginate matrix allows these beads to be washed and reused over multiple cycles. When combined with the resource-recovery strategy of reusing the stripped DB9 dye to dye fresh fabrics [78], the process drastically reduces both raw material costs and waste-disposal expenses, making it highly practical and commercially viable for industrial upscaling.

Furthermore, the potential application of the alginate-immobilized *G. lucidum* matrix in real industrial textile effluents requires a careful consideration of complex matrix effects. Real textile wastewater naturally contains high concentrations of coexisting ions (such as Na^+^, Ca^2+^, Cl^−^, and SO_4_^2−^), surfactants, and auxiliary chemicals, which can cause competitive interference during the biosorption process. Chemically, the presence of background salt ions can shield the positive surface charges on the protonated amino and carboxyl groups of the *G. lucidum* biomass, thereby slightly reducing the electrostatic attraction toward the anionic sulfonate groups of DB9. However, the multi-porous hydrogel structure of the calcium-alginate carrier plays a significant protective role here; it acts as a selective physical barrier that limits the mass transfer of bulky interfering components while allowing smaller dye molecules to diffuse into the active sites. While a direct evaluation using authentic multi-component industrial effluents was not conducted in this work due to technical and practical constraints, the systematic optimization of operational variables established here provides a foundational baseline. Consequently, further investigations involving real industrial matrix validations and continuous multi-stage columns are strictly required before any definitive claims regarding full-scale industrial scalability can be established.

## 4. Discussion

In this study, the performance of free *G. lucidum* biomass was evaluated against alginate-immobilized microbeads to address operational challenges, such as poor mechanical strength and limited reusability, that are traditionally associated with raw fungal biosorbents. The results showed that entrapment inside the alginate matrix increased the DB9 biosorption capacity from 36.798 ± 0.795 mg/g to 89.50 ± 0.01 mg/g. This twofold improvement indicates that the combination of the fungal cell wall and the alginate network creates a more efficient composite system rather than functioning as two separate entities. Recently, it was demonstrated that inactivated *G. lucidum* pellets possess a natural affinity for Direct Blue azo dyes due to their functional surface structure. However, the findings in this study indicate that embedding this biomass in an alginate matrix significantly increases the number of available binding sites [79].

The structural changes observed via FTIR and SEM explain this enhanced performance. The cross-linking process with Ca^2+^ ions introduces abundant carboxyl and hydroxyl groups from the Na-alginate backbone, which complement the amine and phosphate groups naturally found on the *G. lucidum* cell wall. SEM images confirmed that this cross-linking yields a highly porous three-dimensional microstructure. In practical terms, this structure prevents the fungal biomass from aggregating or packing tightly together during operation. By keeping the biosorbent particles discrete and porous, the matrix ensures that dye molecules can easily penetrate the deeper layers of the bead, overcoming the mass-transfer limitations common in raw biomass. A similar mechanism was highlighted in the literature in which Ca-alginate beads served as an excellent porous support structure that improved the stability and interfacial surface area of *G. lucidum* components during textile dye treatment [80].

Equilibrium data fit the Freundlich isotherm, indicating that DB9 biosorption occurs via multilayer binding on a heterogeneous surface. This heterogeneity is logical because the dye molecules are interacting with several distinct types of functional groups on both the fungus and the alginate simultaneously. This behavior aligns with previous studies showing that the biosorption of complex textile dyes onto *G. lucidum* biomass typically involves multiple non-equivalent binding sites. Furthermore, the fit to the pseudo-second-order kinetic model suggests that chemisorption is the rate-limiting step, indicating that uptake relies on chemical interactions, such as electrostatic attraction and hydrogen bonding, rather than on simple physical trapping [81]. The thermodynamic analysis indicated that the process is spontaneous and exothermic. The negative enthalpy (Δ*H* < 0) shows that the bonds formed between DB9 and the biosorbent surface release enough energy to overcome the energy required to dehydrate the dye molecules in the solution.

Reusability experiments demonstrated that both biosorbents retained their performance for at least five biosorption–desorption cycles using ethanol as the desorption agent, while immobilized microbeads exhibited superior structural stability. For practical applicability, the performance of the immobilized microbeads in reusability and continuous column experiments is perhaps the most critical finding for potential industrial scale-up. Raw fungal biomass is notoriously difficult to reuse because it tends to lose mass, break apart, or clog systems during desorption cycles. In our study, the alginate matrix acted as a protective shield, allowing the microbeads to maintain their structural integrity and efficiency over five consecutive cycles using ethanol. This enhancement in structural durability via alginate entrapment is consistent with findings noting that immobilization protects *G. lucidum* structures from chemical and physical degradation during repeated washings [82]. The obtained breakthrough curves confirmed that the immobilized system can sustain efficient removal under dynamic conditions, highlighting its potential for scale-up. Overall, this study demonstrates that alginate-immobilized *G. lucidum* microbeads significantly outperform free biomass in stability, capacity, and operational applicability, offering a promising strategy for developing scalable biosorption systems for azo dye removal.

## Figures and Tables

**Figure 1:**
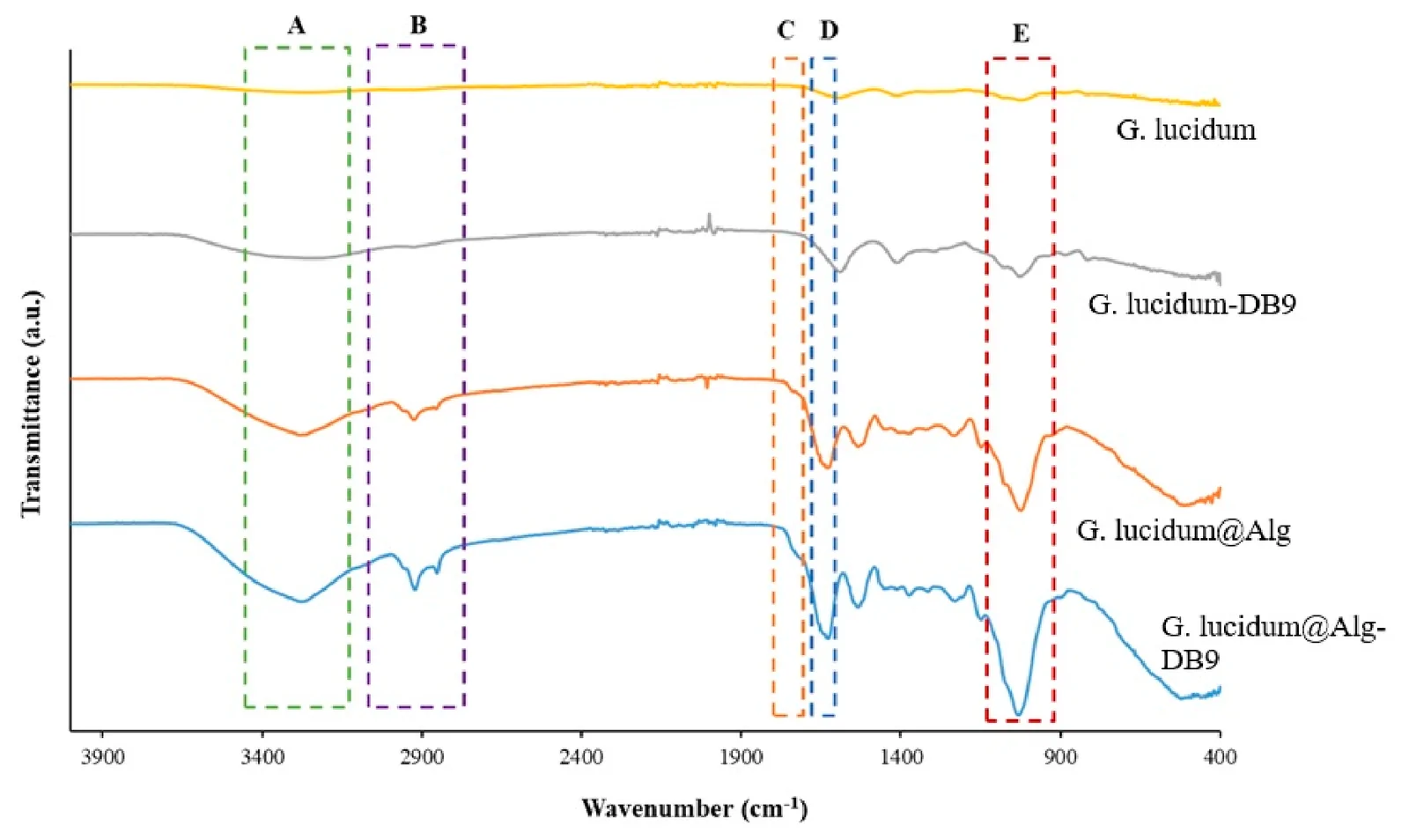
FTIR spectra of *G. lucidum*, alginate-immobilized *G. lucidum* and DB9-loaded counterparts. (A: O-H and N-H stretching vibrations; B: aliphatic C-H stretching vibrations; C: carbonyl (Amide I) stretching vibrations; D: alginate-specific carboxylate stretching region and electrostatic interaction shifts; E: polysaccharide carbohydrate fingerprint vibrations of the adsorbed DB9).

**Figure 2:**
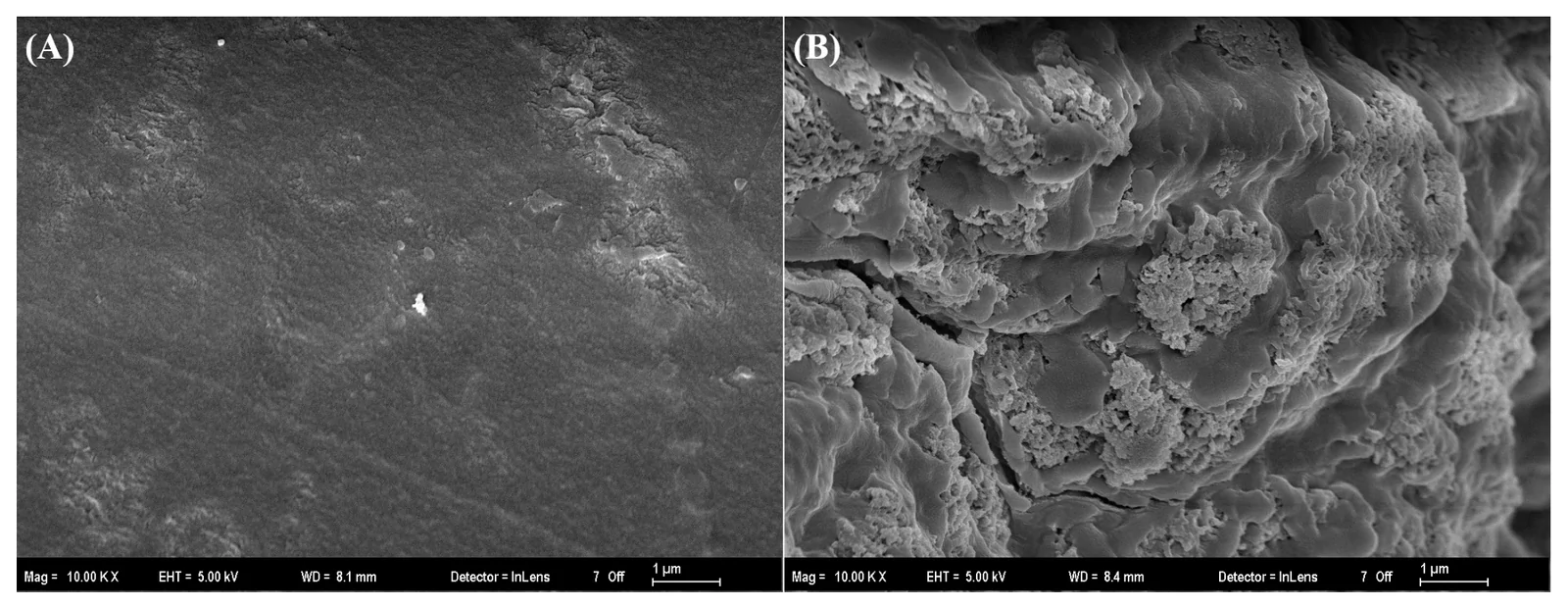
SEM images of (**A**) alginate and (**B**) alginate-immobilized *G. lucidum* microbeads.

**Figure 3:**
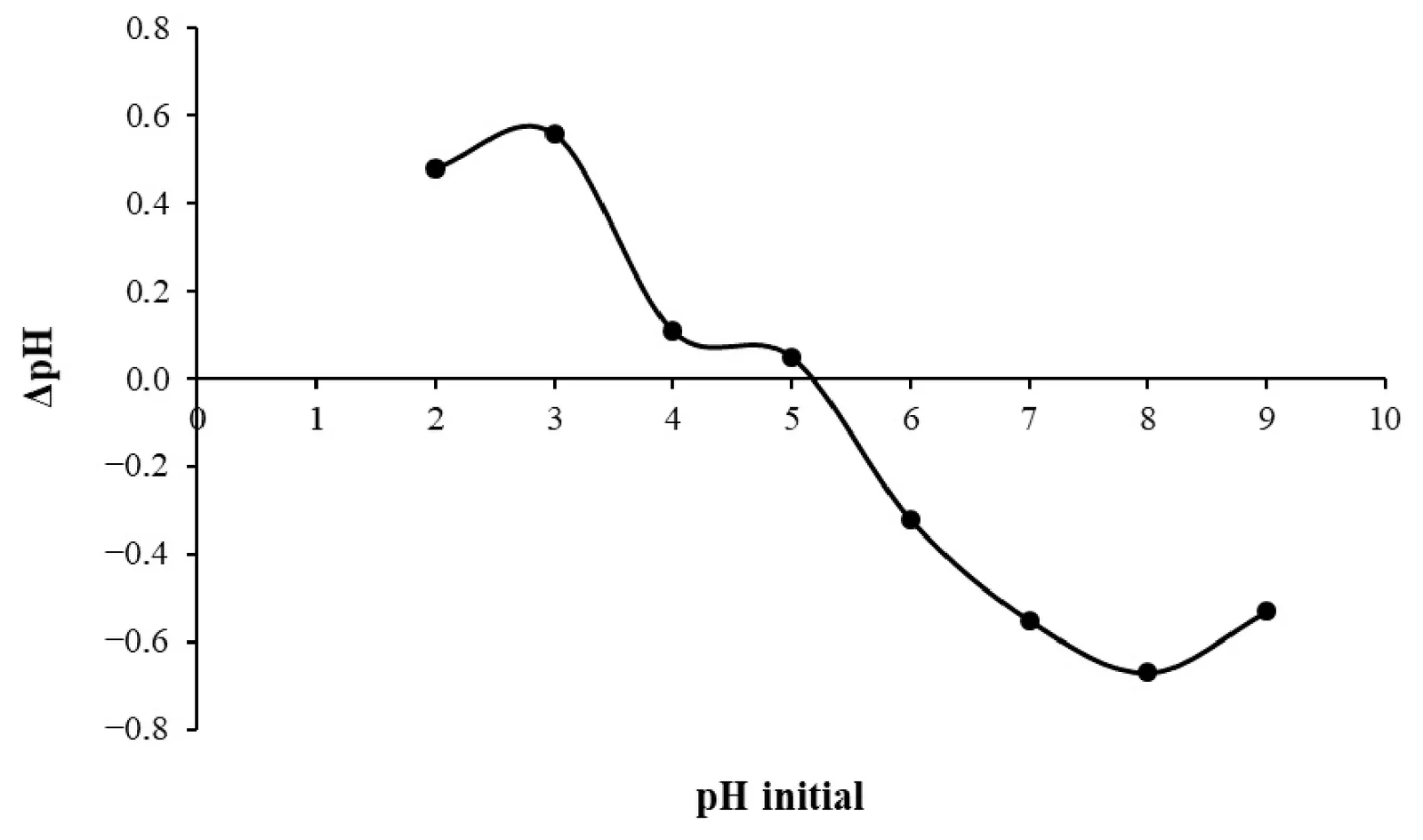
pH at the point of zero charge (*pH_pzc_*) of the alginate-immobilized *G. lucidum* microbeads.

**Figure 4:**
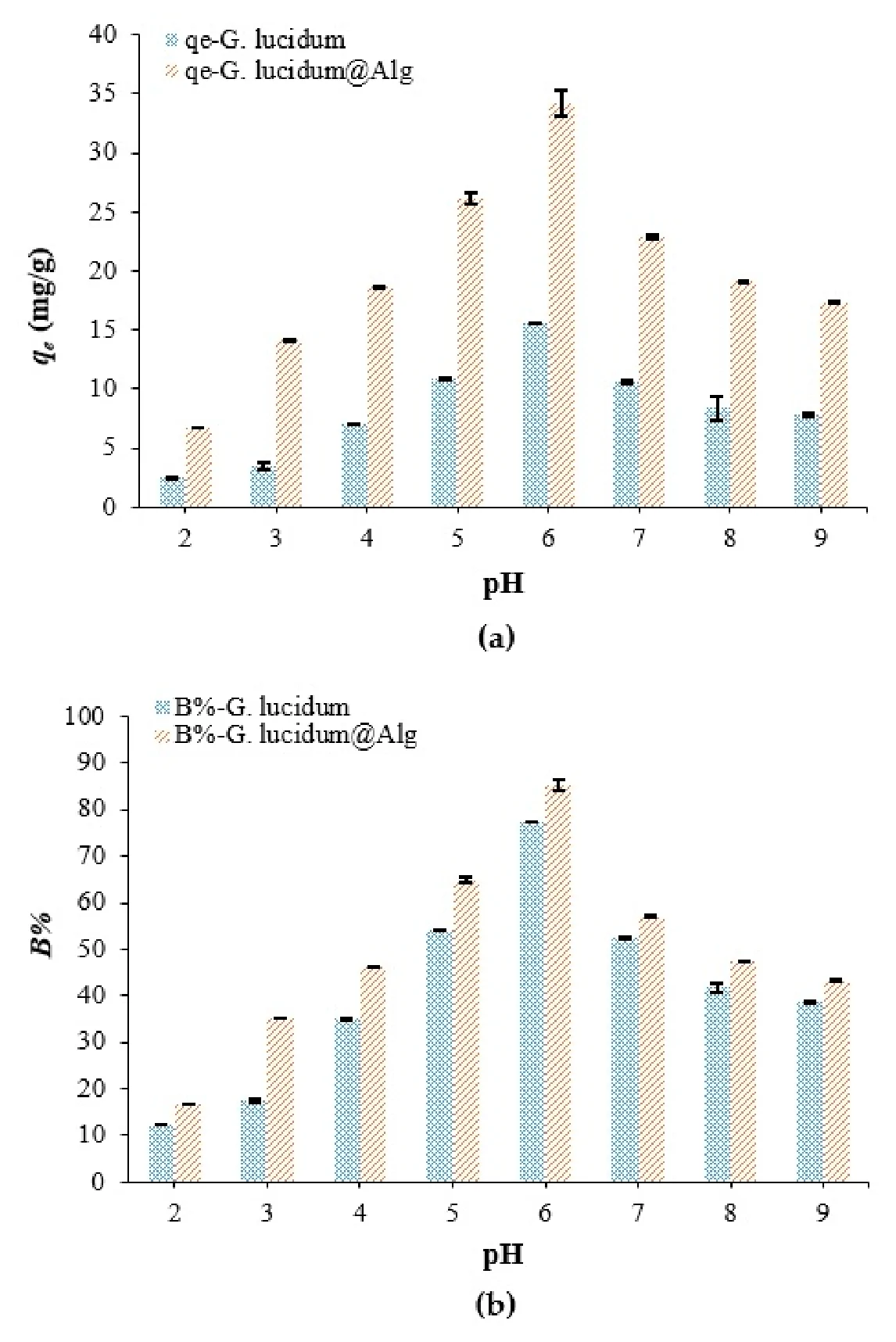
Effect of initial solution pH on (**a**) biosorption capacity (*q_e_*), and (**b**) biosorption percentage (*B*%). (Experimental conditions: *C_i_*: 100 mg/L; *T*: 25 °C, contact time: 180 min).

**Figure 5:**
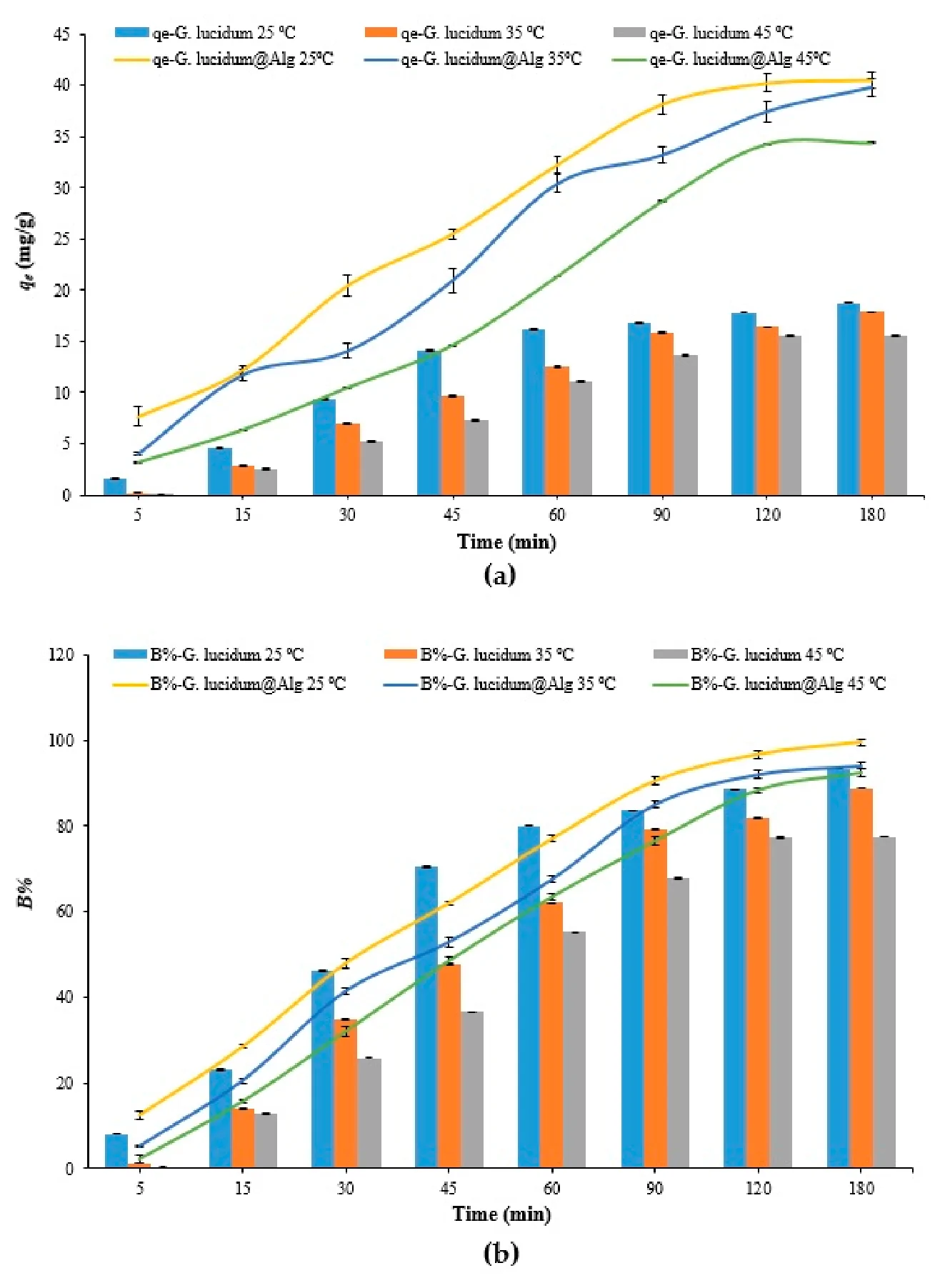
Effect of temperature at different contact times on (**a**) biosorption capacity (*q_e_*), and (**b**) removal percentage (*B*%). (Experimental conditions: *C_i_*: 100 mg/L; pH:6.0; *T*: 25, 35, and 45 °C, contact time: 5–180 min).

**Figure 6:**
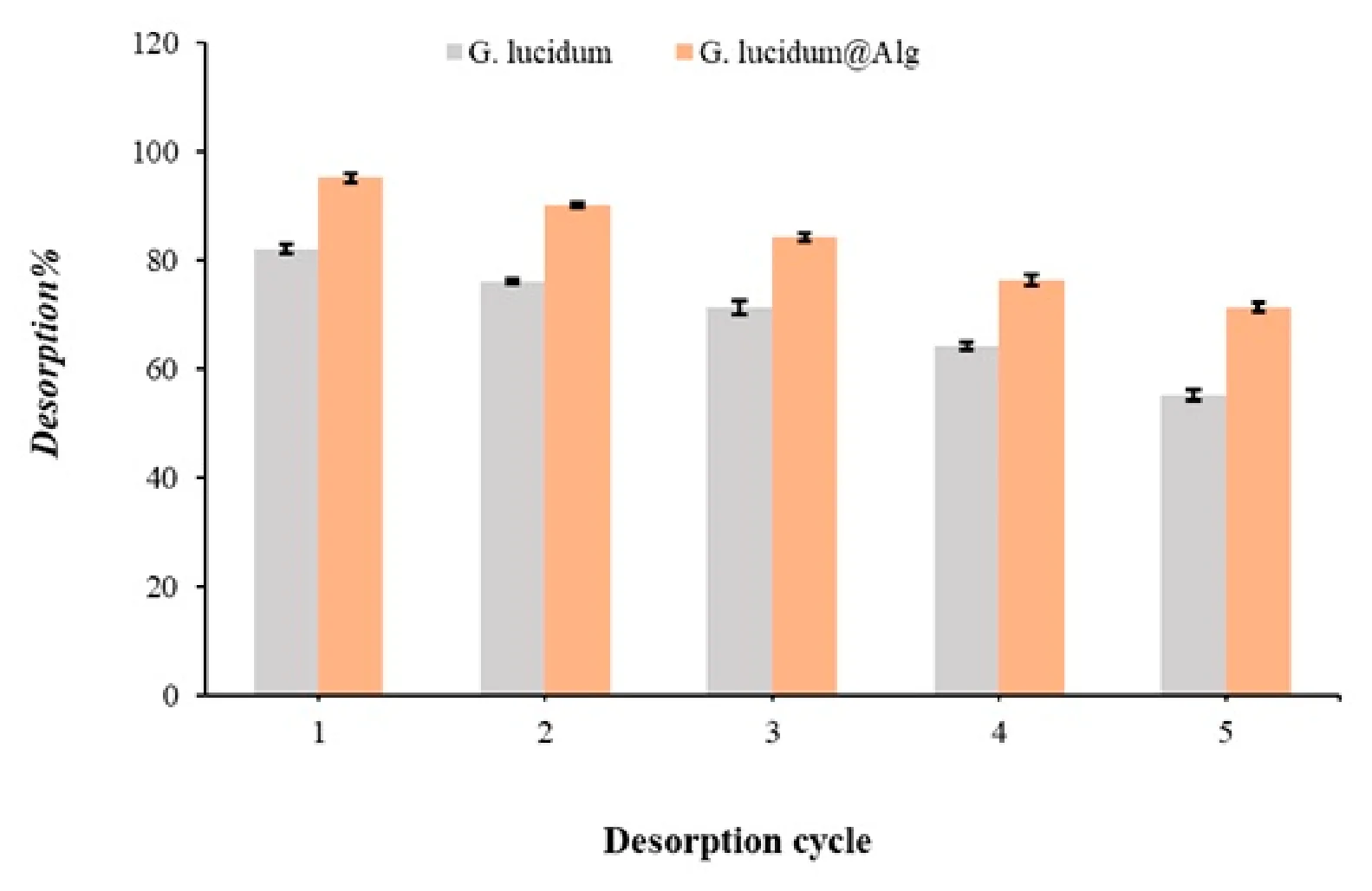
Reusability and desorption cycles of the biosorbents over multiple runs (eluent: ethanol).

**Figure 7:**
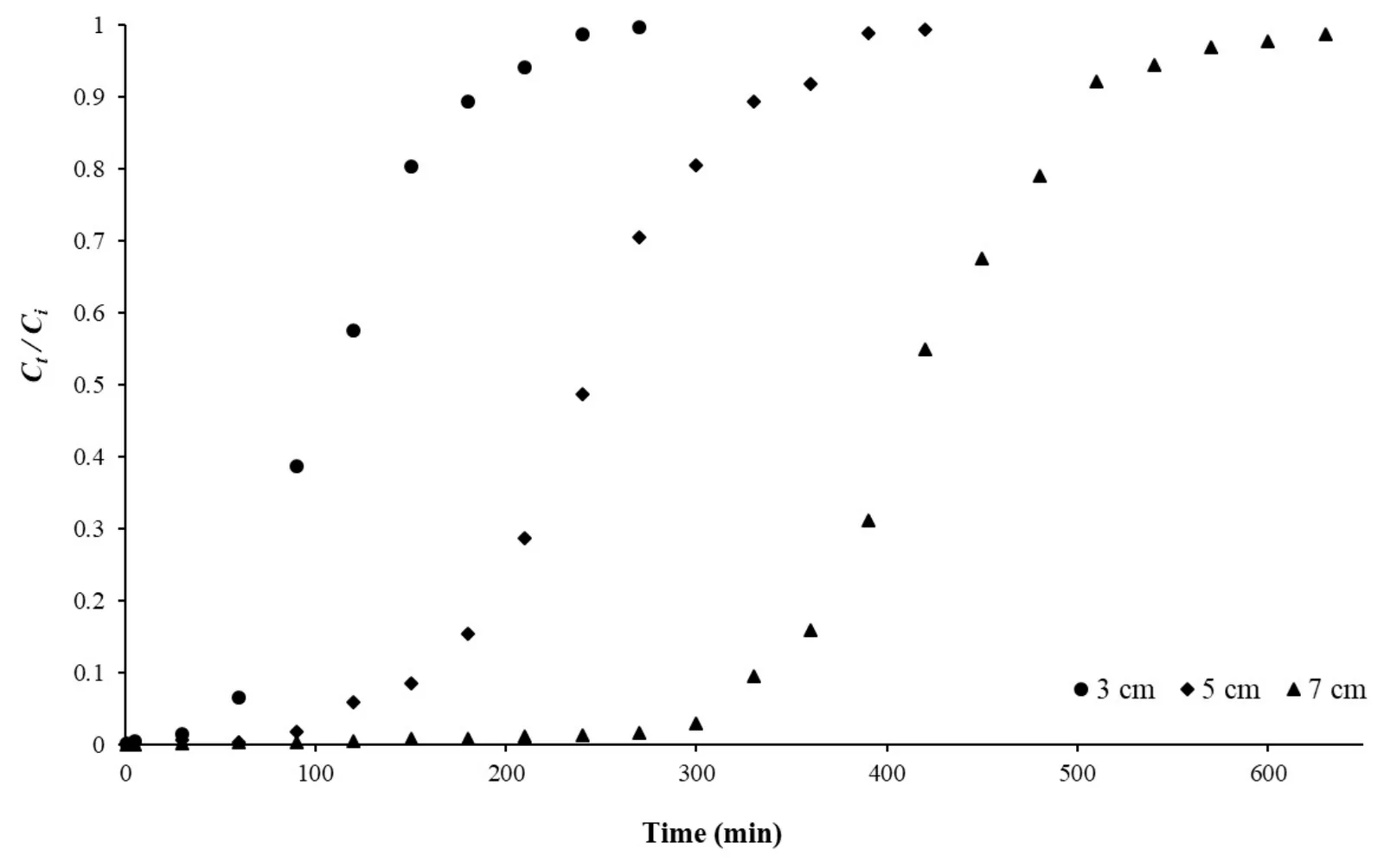
Breakthrough curves obtained at different bed heights.

**Figure 8:**
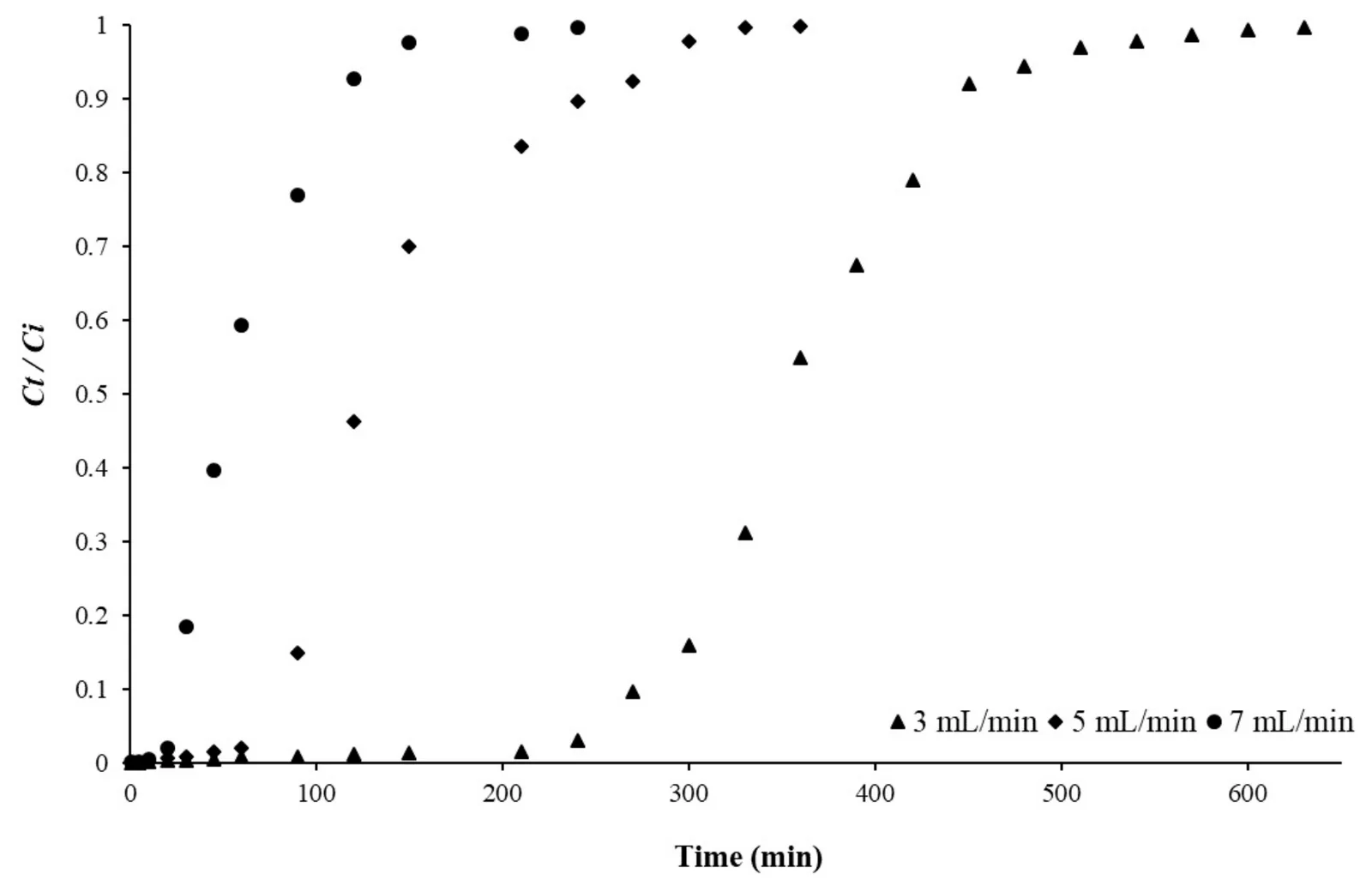
Breakthrough curves obtained at different flow rates.

**Figure 9:**
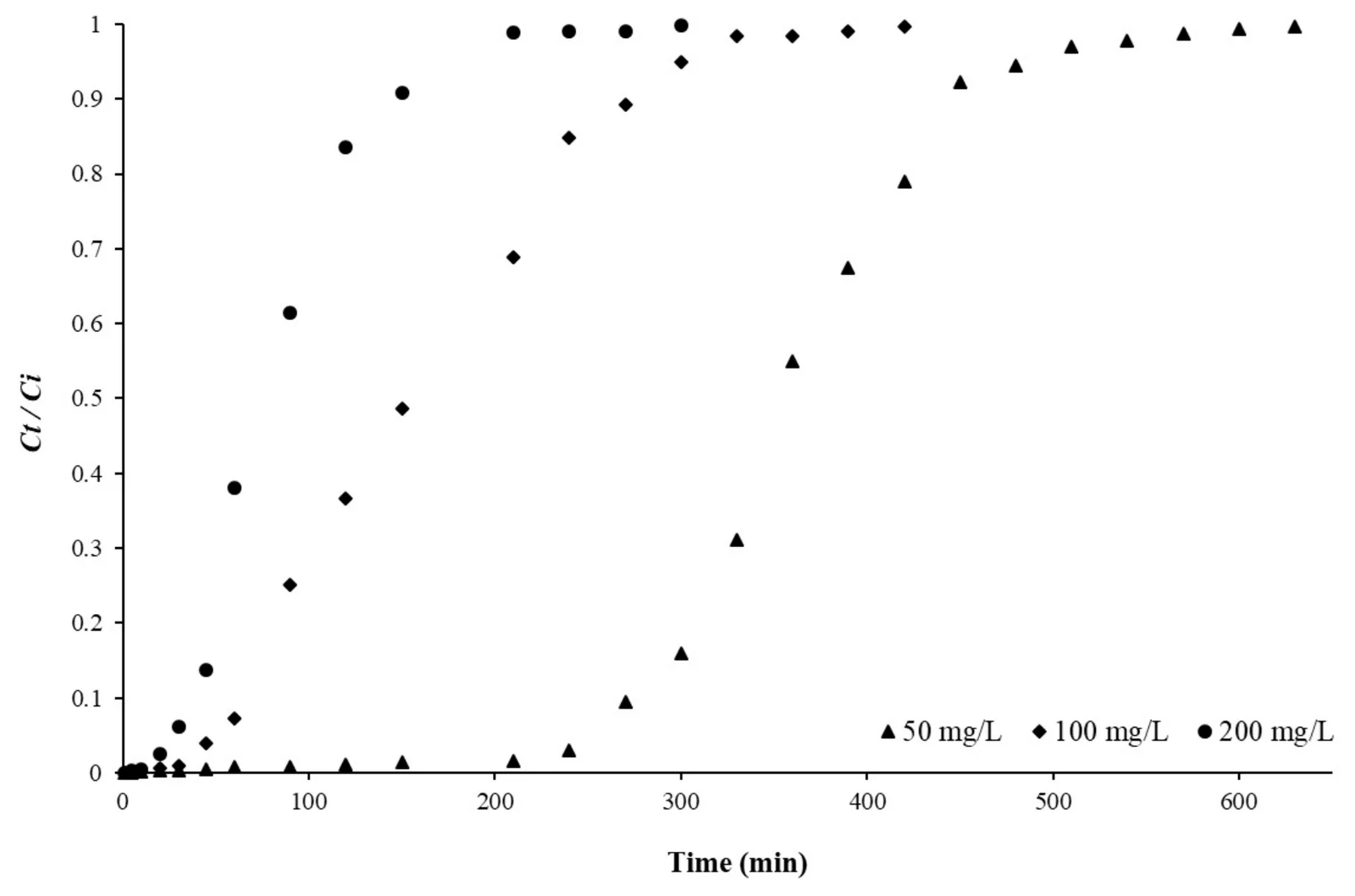
Breakthrough curves obtained at different initial DB9 concentrations.

**Table 1:** Gel fraction (*F_gel_*), water content (*C_w_*), and swelling ratios (*Swelling*%) of microbeads prepared in different formulations.

	0.5 g of *G. lucidum*				
	Alginate Amount (*w*/*v*)				
	1%	2%	3%	4%	5%
*F_gel_*	98.67	98.75	99.86	99.15	98.89
*C_w_*	16.52	17.23	18.37	16.62	16.56
*Swelling*%	85.48	87.29	93.61	89.58	83.93

**Table 2:** Isotherm parameters for the biosorption of DB9 dye onto *G. lucidum* biomass and alginate-immobilized *G. lucidum* microbeads.

	*G. lucidum*			Alginate-Immobilized *G. lucidum* Microbeads		
T (K)	298	308	318	298	308	318
Langmuir						
*K_L_* (L/mg)	0.061	0.244	0.059	0.294	0.718	0.534
*Q_L_* (mg/g)	2.894	2.576	0.769	4.239	9.643	19.841
*R* ^2^	0.767	0.884	0.747	0.917	0.985	0.996
*R_L_*	0.308	0.107	0.369	0.014	0.016	0.008
Freundlich						
*K_F_* (L/mg)	0.016	1.726	7.15 × 10^−5^	5.027	7.325	6.012
*n*	0.458	0.982	0.271	0.129	0.166	0.493
*R* ^2^	0.966	0.988	0.931	0.995	0.990	0.982
Dubinin–Radushkevich (D-R)						
*K_D-R_* × 10^−3^ (mol^2^/J^2^)	7 × 10^−5^	2 × 10^−6^	2 × 10^−4^	1 × 10^−5^	5 × 10^−7^	7 × 10^−7^
*Q_D-R_* (mg/g)	61.295	21.568	48.043	27.841	28.651	29.621
*E_fe_* (kJ/mol)	0.05	0.5	0.085	0.845	1	0.707
*R* ^2^	0.981	0.960	0.965	0.901	0.918	0.893

**Table 3:** Kinetic parameters for the biosorption process of DB9 dye using *G. lucidum* biomass and alginate-immobilized *G. lucidum* microbeads.

		Pseudo-First Order			Pseudo-Second Order			Intraparticular	
	T (K)	*q_e_*	*k*_1_ (1/min)	*R* ^2^	*q_e_*	*k*_2_ ((g/mg)/min)	*R* ^2^	*k_id_*	*R* ^2^
*G. lucidum*	298	17.771	0.024	0.982	21.263	9.35 × 10^−4^	0.961	1.615	0.861
	308	24.757	0.027	0.979	18.553	3.11 × 10^−4^	0.863	1.708	0.941
	318	19.342	0.024	0.927	13.495	2.67 × 10^−4^	0.842	1.559	0. 939
Alginate-immobilized *G. lucidum* microbeads	298	17.624	0.020	0.932	22.573	10.48 × 10^−4^	0.982	1.651	0.919
	308	28.695	0.029	0.959	20.877	5.38 × 10^−4^	0.958	1.719	0.941
	318	20.695	0.020	0.919	16.695	2.79 × 10^−4^	0.876	1.653	0.951

**Table 4:** Dye removal by various biosorbents.

Dye	Biosorbent	Biosorption%	Isotherm	Kinetic	References
Acid Blue 161	*G. lucidum*	65.83	Langmuir	-	[17]
Reactive Red 43		27.77			
Safranine T	*G. lucidum*	82	Langmuir and Freundlich	Pseudo second order	[68]
Malachite Green		92			
Methylene Blue		92			
Simulated effluent	*Trametes* sp.	89.47	Langmuir	Avrami fractional order	[69]
Congo Red	*Aspergillus niger*	84.37	Langmuir	Pseudo second order	[40]
Yellow 4G	*Morchella esculenta* immobilized alginate-PVA	96.24	-	-	[70]
Congo Red	*Neurospora crassa* immobilized alginate beads	89.32	Langmuir	Pseudo second order	[35]
Alizarin Red S	*Aspergillus niger*/alginate beads	97.40	Langmuir	Pseudo second order and intraparticle diffusion	[71]
Direct Blue 9	*G. lucidum*	93.31 ± 0.05	Freundlich	Pseudo second order	Current study
	Alginate-immobilized *G. lucidum* microbeads	99.54 ± 0.76			

**Table 5:** Thermodynamic parameters calculated for the biosorption of DB9 dye using *G. lucidum* biomass and alginate-immobilized *G. lucidum* microbeads.

	*G. lucidum*			Alginate-Immobilized *G. lucidum* Microbeads		
*T* (K)	298	308	318	298	308	318
Δ*G*° (kJ/mol)	−105.273	−106.912	−108.550	−134.167	−136.366	−138.565
Δ*S°* (J/mol K)	163.836			219.905		
Δ*H*° (kJ/mol)	−56.450			−68.635		

## Data Availability

The original contributions presented in this study are included in the article. Further inquiries can be directed to the corresponding author.

## Supplementary Materials

The following supporting information can be downloaded at https://www.mdpi.com/article/10.3390/polym18192317/s1: Figure S1. Effect of various concentrations on (a) biosorption capacity (*q_e_*), and (b) biosorption percentage (*B*%). (Experimental conditions: pH:6.0; T: 25 °C, contact time: 120 min). Figure S2. Effect of various concentrations on biosorption capacity (*q_e_*) for *G. lucidum* (blue bar), alginate immobilized *G. lucidum* (orange bar), and alginate microbeads (grey bar). (Experimental conditions: pH:6.0; T: 25 °C, contact time: 120 min). Figure S3. Optical images of alginate-immobilized *G. lucidum* microbeads (A) before biosorption, (B) after biosorption, and (C) after desorption processes. Table S1. Two-Way ANOVA results comparing the equilibrium biosorption capacity (qe) of free and alginate-immobilized *G. lucidum* biomass across various pH levels. Table S2. Two-Way ANOVA results for the equilibrium biosorption capacity (*q_e_*) of free and alginate-immobilized *G. lucidum* biomass at the final equilibrium time (180 min) under different temperatures. Table S3. Two-Way ANOVA results evaluating the desorption efficiency (%) of free and alginate-immobilized *G. lucidum* biomass over five regeneration cycles. Table S4. Two-Way ANOVA results for the maximum equilibrium biosorption capacity (*q_e_* at 200 mg/L) of free and alginate-immobilized *G. lucidum* biomass under different temperatures. Table S5. One-Way ANOVA results comparing the maximum equilibrium biosorption capacity (*q_e_* at 200 mg/L and 25 °C) among free *G. lucidum*, alginate-immobilized *G. lucidum* (*G. lucidum*@Alg) microbeads, and pure alginate (Alg) microbeads.

## Abbreviations

The following abbreviations are used in this manuscript:

*B*%	Biosorption percentage
DB9	Direct Blue 9
Δ*G*°	Free energy change
Δ*H*°	Enthalpy change
Δ*S*°	Entropy change
*C*_0_	initial dye concentration
*C_e_*	equilibrium dye concentration
*E_fe_*	Free energy of biosorption
*F_gel_*	gel fraction
FTIR	Fourier transform infrared
*k*_1_	Equilibrium rate constant of pseudo-first order
*k*_2_	Pseudo-second-order rate constant
*K_D-R_*	D-R constant
*K_F_*	Biosorption capacity of Freundlich isotherm model
*K_L_*	Biosorption capacity of Langmuir isotherm model
*m*	amount of biosorbent
*MB*	Methylene Blue
*n*	Freundlich isotherm constant
*Q_b_*	biosorbed amount
*Q_d_*	desorbed amount
*Q_D-R_*	Maximum biosorption
*q_e_*	biosorption capacity
*q_t_*	Amount of dye biosorbed time *t*
*R*	Universal gas constant
SEM	Scanning Electron Microscopy
*T*	Temperature
*V*	total volume of the dye solution
*ε*	Polanyi potential
