# Supplementary material for: Enhanced Azo Dye Removal via Alginate-Immobilized Ganoderma lucidum: From Batch Experiments to Continuous Column Studies
Source: Polymers (Basel). 2026 Sep 22;18(19):2317. doi: 10.3390/polym18192317 (PMC13666684; doi:10.3390/polym18192317)
Supplement: Supplementary file 1 [file polymers-18-02317-s001.zip › polymers-4547801-supplementary.pdf]

Supplementary Information

Enhanced azo dye removal via alginate-immobilized *Ganoderma lucidum*: From batch experiments to continuous column studies

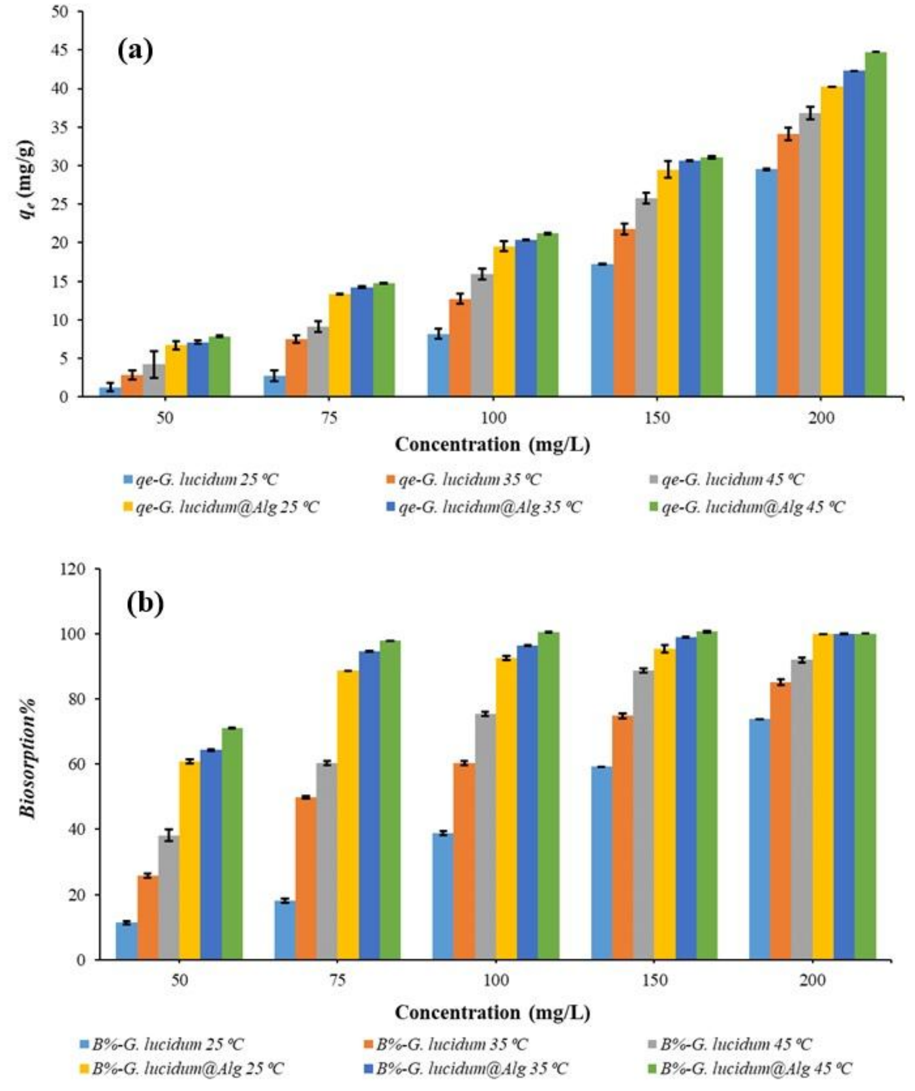

Figure S1. Effect of various concentrations on **(a)** biosorption capacity (q_e), and **(b)** biosorption percentage (*B*%). (Experimental conditions: pH:6.0; T: 25 ^{0}C, contact time: 120 min).

To evaluate the simultaneous effects of biomass form and temperature on the maximum biosorption capacity, a Two-Way ANOVA was performed on the equilibrium data at the highest initial concentration (200 mg/L). The statistical analysis indicated that the immobilization process exerted a highly significant positive effect on the maximum q_e ($F_{(1,12)}$ = 4461, $p < 0.001$). Although temperature variations also significantly influenced the maximum capacity ($F_{(2,12)}$ = 902, $p < 0.001$) alongside a significant interaction effect ($F_{(2,12)}$ = 257, $p < 0.001$). As a result (Table S4), the alginate-immobilized *G. lucidum* microbeads maintained an overwhelming statistical superiority over the free biomass form across all tested temperatures.

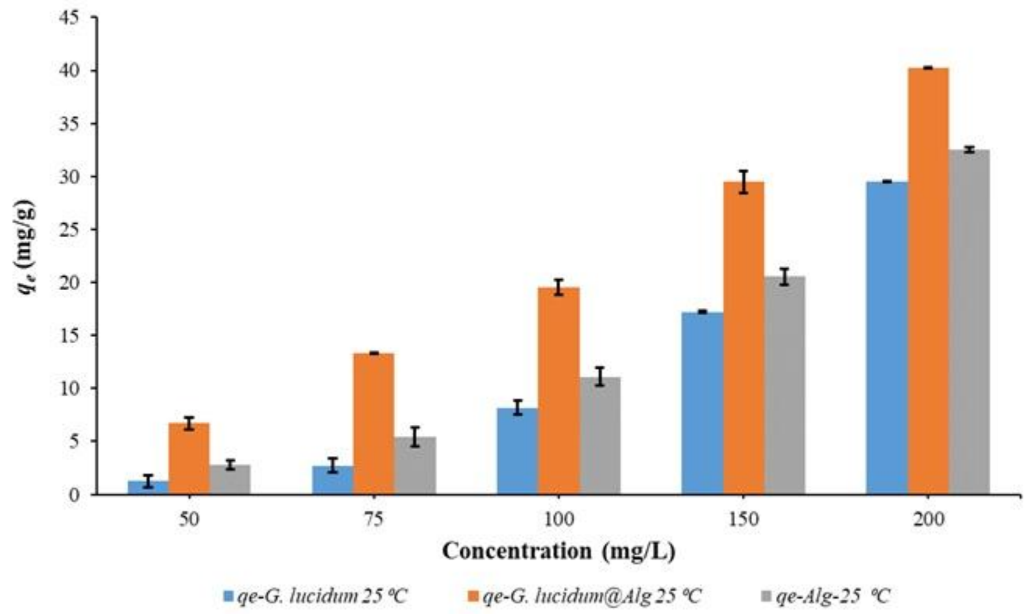

Figure S2. Effect of various concentrations on biosorption capacity (q_e) for *G. lucidum* (blue bar), alginate immobilized *G. lucidum* (orange bar), and alginate microbeads (grey bar). (Experimental conditions: pH:6.0; T: 25 ^{0}C, contact time: 120 min).

To mathematically distinguish the performance of the alginate-immobilized *G. lucidum* microbeads frm pure alginate (Alg) microbeads and free biomass (*G. lucidum*), a One-Way ANOVA was applied to the maximum equilibrium capacity (q_e at 200 mg/L). The statistical analysis confirmed that the biosorbent type plays a decisive role in the process ($F_{(2,6)}$ = 1063, $p < 0.001$). Furthermore, the post-hoc Tukey test demonstrated that the alginate-immobilized *G. lucidum* microbeads possess a highly significant advantage over both the free biomass and the blank alginate matrix ($p < 0.001$) (Table S5). This statistical backing rigorously validates that the enhanced biosorption performance stems from the synergistic integration of the biomass within the polymeric framework rather than the matrix itself.

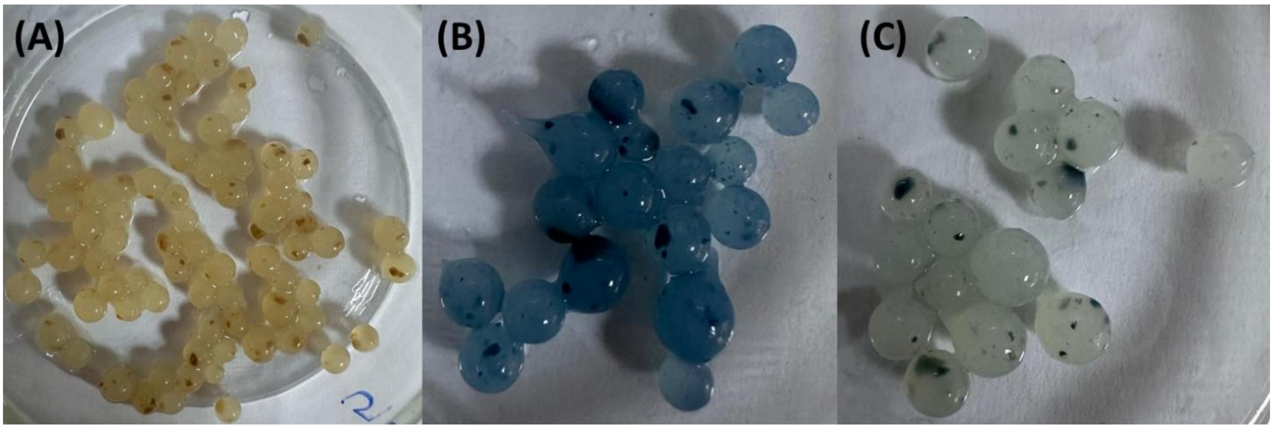

Figure S3. Optical images of alginate-immobilized *G. lucidum* microbeads (A) before biosorption, (B) after biosorption, and (C) after desorption processes.

Table S1. Two-Way ANOVA results comparing the equilibrium biosorption capacity (qe) of free and alginate-immobilized *G. lucidum* biomass across various pH levels.

	Sum of Squares	df	Mean Square	F	p
Biomass	1634.05	1	1634.0451	32686	< 0.001
pH	1611.50	7	230.2141	4605	< 0.001
Biomass * pH	193.93	7	27.7039	554	< 0.001
Residuals	1.60	32	0.0500		

Table S2. Two-Way ANOVA results for the equilibrium biosorption capacity (q_e) of free and alginate-immobilized *G. lucidum* biomass at the final equilibrium time (180 min) under different temperatures.

	Sum of Squares	df	Mean Square	F	p
Biomass	1967.171	1	1967.1710	33140.5	< 0.001
Temperature	69.946	2	34.9730	589.2	< 0.001
Biomass * Temperature	8.825	2	4.4127	74.3	< 0.001
Residuals	0.712	12	0.0594		

Table S3. Two-Way ANOVA results evaluating the desorption efficiency (%) of free and alginate-immobilized *G. lucidum* biomass over five regeneration cycles.

	Sum of Squares	df	Mean Square	F	p
Biomass	1436.29	1	1436.288	12910.3	< 0.001
Cycle	2466.22	4	616.554	5542.0	< 0.001
Biomass * Cycle	11.45	4	2.863	25.7	< 0.001
Residuals	2.23	20	0.111		

Table S4. Two-Way ANOVA results for the maximum equilibrium biosorption capacity (q_e at 200 mg/L) of free and alginate-immobilized *G. lucidum* biomass under different temperatures.

	Sum of Squares	df	Mean Square	F	p
Biomass	464.52	1	464.515	4461	< 0.001
Temperature	187.81	2	93.907	902	< 0.001
Biomass * Temperature	53.55	2	26.773	257	< 0.001
Residuals	1.25	12	0.104		

Table S5. One-Way ANOVA results comparing the maximum equilibrium biosorption capacity (q_e at 200 mg/L and 25 ^{0}C) among free *G. lucidum*, alginate-immobilized *G. lucidum* (*G. lucidum*@Alg) microbeads, and pure alginate (Alg) microbeads.

One-Way ANOVA (Fisher's)

	F	df1	df2	p
qe	1063	2	6	< 0.001

Tukey Post-Hoc Test – qe

		G. lucidum	*G. lucidum*@Alg	Alg
G. lucidum	Mean difference	-	-11.0	-3.19
	p-value	-	< 0.001	< 0.001
G. lucidum@Alg	Mean difference		-	7.84
	p-value		-	< 0.001
Alg	Mean difference			-
	p-value			-
